# Late‐onset retinal degeneration pathology due to mutations in CTRP5 is mediated through HTRA1

**DOI:** 10.1111/acel.13011

**Published:** 2019-08-05

**Authors:** Anil Chekuri, Katarzyna Zientara‐Rytter, Angel Soto‐Hermida, Shyamanga Borooah, Marina Voronchikhina, Pooja Biswas, Virender Kumar, David Goodsell, Caroline Hayward, Peter Shaw, Chloe Stanton, Donita Garland, Suresh Subramani, Radha Ayyagari

**Affiliations:** ^1^ Shiley Eye Institute University of California San Diego San Diego CA USA; ^2^ Division of Biological Sciences University of California San Diego San Diego CA USA; ^3^ Integrative Structural and Computational Biology (ISCB) Scripps Research Institute San Diego CA USA; ^4^ Medical Research Council Human Genetics Unit, Medical Research Council Institute of Genetics and Molecular Medicine University of Edinburgh Edinburgh UK; ^5^ Massachusetts Eye and Ear Infirmary, Department of Ophthalmology Harvard Medical School Boston MA USA

**Keywords:** age‐related macular degeneration, CTRP5, drusen, ECM remodeling, HTRA1, L‐ORD, sub‐RPE deposits

## Abstract

Late‐onset retinal degeneration (L‐ORD) is an autosomal dominant macular degeneration characterized by the formation of sub‐retinal pigment epithelium (RPE) deposits and neuroretinal atrophy. L‐ORD results from mutations in the C1q‐tumor necrosis factor‐5 protein (CTRP5), encoded by the *CTRP5/C1QTNF5 gene*. To understand the mechanism underlying L‐ORD pathology, we used a human cDNA library yeast two‐hybrid screen to identify interacting partners of CTRP5. Additionally, we analyzed the Bruch's membrane/choroid (BM‐Ch) from wild‐type (*Wt*), heterozygous S163R *Ctrp5* mutation knock‐in (*Ctrp5^S163R/wt^*), and homozygous knock‐in *(Ctrp5^S163R/S163R^*) mice using mass spectrometry. Both approaches showed an association between CTRP5 and HTRA1 via its C‐terminal PDZ‐binding motif, stimulation of the HTRA1 protease activity by CTRP5, and CTRP5 serving as an HTRA1 substrate. The S163R‐CTRP5 protein also binds to HTRA1 but is resistant to HTRA1‐mediated cleavage. Immunohistochemistry and proteomic analysis showed significant accumulation of CTRP5 and HTRA1 in BM‐Ch of *Ctrp5^S163R/S163R^* and *Ctrp5^S163R/wt^* mice compared with *Wt*. Additional extracellular matrix (ECM) components that are HTRA1 substrates also accumulated in these mice. These results implicate HTRA1 and its interaction with CTRP5 in L‐ORD pathology.

## 1 INTRODUCTION

L‐ORD is an autosomal dominant macular degeneration resulting from mutations in the *CTRP5/C1QTNF5* gene, encoding C1q‐tumor necrosis factor‐related protein 5. Clinically, it is characterized by the onset of sub‐retinal drusen‐like deposits and abnormal dark adaptation from the 5th decade of life, and chorioretinal atrophy and choroidal neovascularization in the 6th decade, progressing to significant vision loss (Ayyagari et al., 2000, 2005). L‐ORD has phenotypic similarities with the common condition and age‐related macular degeneration (AMD) (Ayyagari et al., 2000, 2005; Borooah, Collins, Wright, & Dhillon, 2009; Cukras, Ayyagari, Wong, & Sieving, 2015). A heterozygous missense mutation, S163R, in the CTRP5 protein was originally shown to segregate with L‐ORD in several families (Ayyagari et al., 2005; Hayward et al., 2003). More recently, three additional mutations in the C1q domain of CTRP5 were identified in patients with L‐ORD (Borooah et al., 2018; Stanton et al., 2017). Despite the identification of these mutations, little is known about the mechanism underlying L‐ORD pathology.

*CTRP5* is highly expressed in RPE cells as a bicistronic transcript with membrane frizzled‐related protein (*MFRP)* that is encoded by another retinal disease‐causing gene (Mandal, Vasireddy, Jablonski, et al., 2006; Mandal, Vasireddy, Reddy, et al., 2006). *CTRP5* is a 25 KDa protein belonging to the C1q tumor necrosis factor superfamily of 10 structurally similar, but functionally diverse, proteins (Ghai et al., 2007; Kouser et al., 2015). Proteins in this family have an N‐terminal signal peptide, a short variable region followed by a short‐chain collagen‐like domain and a C‐terminal C1q domain. The C1q domain is essential for trimerization and the subsequent formation of higher order multimers (Tu & Palczewski, 2012). CTRP5, a secreted protein, forms trimers as well as octadecamers (bouquet‐like structures); the multimers are predicted to be the functional form of the protein (Stanton et al., 2017; Tu & Palczewski, 2014). The S163R mutation in CTRP5 is predicted to alter its structure and consequently its function (Shu et al., 2006; Tu & Palczewski, 2012, 2014). In addition, the S163R mutation impairs its secretion (Mandal, Vasireddy, Reddy, et al., 2006; Shu et al., 2011).

The current study is focused on understanding the role of the S163R CTRP5 mutation in L‐ORD pathology using two global discovery methods, a yeast two‐hybrid (Y2H) screen to identify CTRP5‐interaction partners and a proteomic approach to identify altered proteins in the Bruch's membrane/choroid (BM‐Ch) tissue involved in disease pathology. Mice with homozygous or heterozygous S163R CTRP5 mutations served as models to study the disease pathology.

## 2 RESULTS

### 2.1 Identification of HTRA1 as a CTRP5‐binding partner using the Y2H system

**Figure 1:**
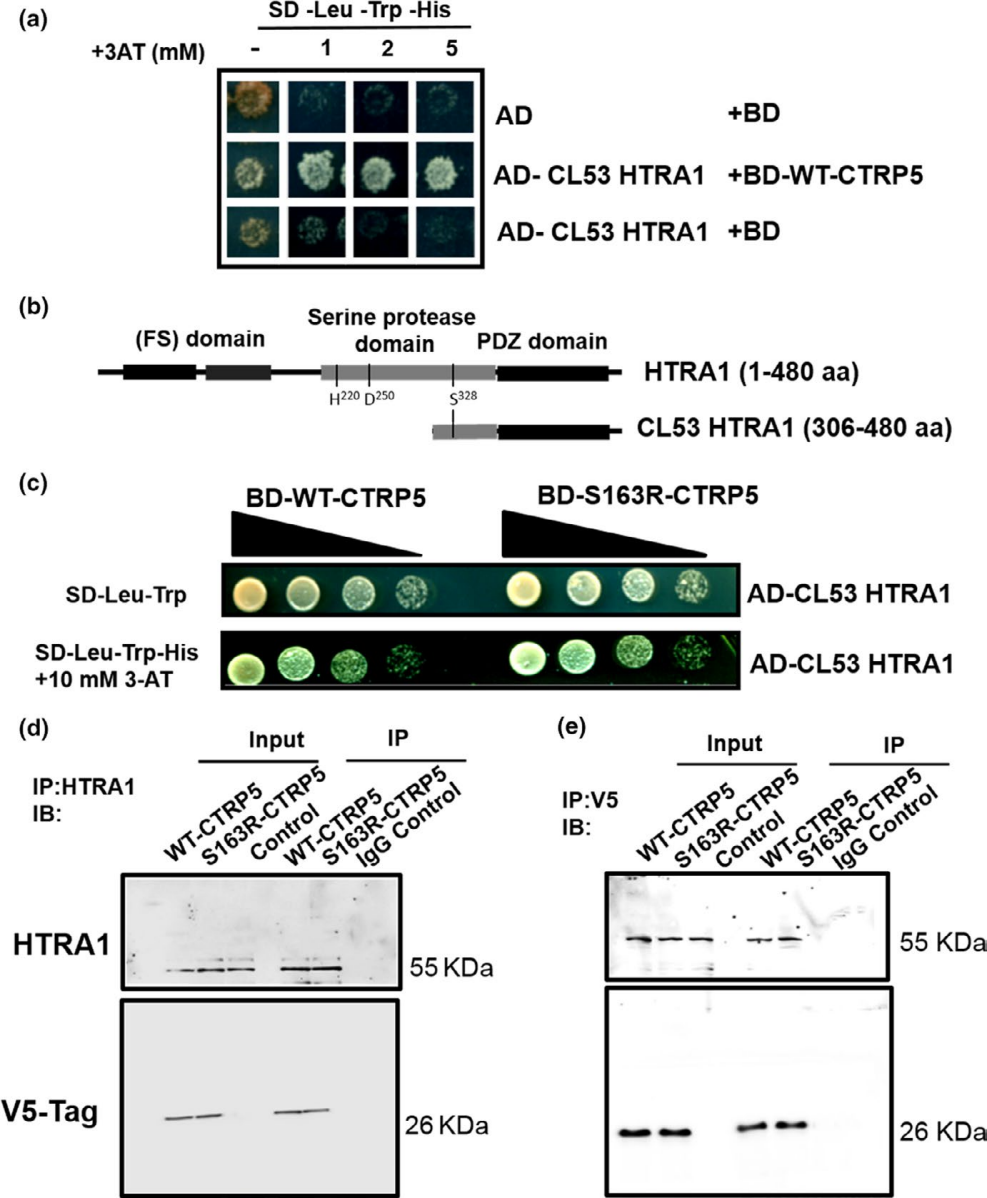
CTRP5 interacts with HTRA1. (a) Clone 53 (CL53) containing a partial sequence of HTRA1 was identified as a CTRP5 interactor during Y2H screening of a normalized human cDNA library cloned into a prey vector, pGADT7‐RecAB, containing the activation domain (AD) of GAL4. Full‐length WT‐CTRP5 fused to the binding domain (BD) of GAL4 (BD‐WT‐CTRP5) was used as bait. An empty bait plasmid was used as a control to exclude false‐positive interactions. Yeast cells carrying the proper combinations of bait and prey plasmids were plated on semi‐selective (SD‐Leu‐Trp, i.e., selective only for the bait and prey plasmids) and selective (SD‐Leu‐Trp‐His, i.e., selective for the bait and prey plasmids and the protein–protein interaction) media supplemented with 3‐AT (1, 2, and 5 mM). (b) Schematic of full‐length HTRA1 with domain organization in comparison with the corresponding sequence (aa 306–480) of clone 53 obtained from Y2H library screening for CTRP5 interactors. The follistatin (FS) domain is a combination of the IGF‐binding protein (IGFBP) and the Kazal‐type serine protease inhibitor (KI) domains.The position of HTRA1 catalytic triad (Ser328, His220, and Asp250) is indicated. (c) HTRA1 interacts with both WT‐CTRP5 and S163R‐CTRP5 with similar binding affinities in the Y2H assay. Yeast cells containing AD‐CL53 HTRA1 (306–480aa) and BD‐WT‐CTRP5 or BD‐S163R‐CTRP5 constructs were plated at serial 10‐fold dilutions with a starting concentration of OD_600_ of 0.8 on semi‐selective (SD‐Leu‐Trp) and on selective (SD‐Leu‐Trp‐His) media supplemented with 10 mM 3‐AT. (d) Co‐Immunoprecipitation assay for confirmation of CTRP5 and HTRA1 interaction. Lysates from ARPE‐19 cells overexpressing WT‐CTRP5‐V5 or S163R‐CTRP5‐V5 constructs were used for immunoprecipitation (IP) using HTRA1 antibody. Immunoprecipitates were resolved on 10% SDS‐PAGE followed by immunoblotting (IB). Blots were probed with anti‐V5 and HTRA1 antibodies. Normal rabbit IgG (IgG control) was used as a negative control to determine the antibody specificity. Lysates of untransfected cells were used as control for overexpression. Input has 10% of total lysates. Cells transfected with the empty vector were used as a transfection control in the experiment. (e) Reverse co‐immunoprecipitation was performed using lysates from cells overexpressing WT‐CTRP5‐V5 or S163R‐CTRP5‐V5 constructs. Immunoprecipitation was done using anti‐V5 antibody, and proteins were detected in immunoblots using anti‐V5 and HTRA1 antibodies

In order to understand the physiological role of CTRP5 and to identify novel binding partners, the Matchmaker Gold Y2H system was used (Chien, Bartel, Sternglanz, & Fields, 1991; Fields & Song, 1989). Our preliminary experiments showed that full‐length WT‐CTRP5 was expressed in yeast without auto‐activation and bound to its known interactor, MFRP (Mandal, Vasireddy, Jablonski, et al., 2006; Tu & Palczewski, 2014), as well as to itself (Wong et al., 2008) (Figure S1). This result proved that CTRP5 is a good candidate for the Y2H screen as it is not toxic to yeast and recognizes its binding partners without causing auto‐activation of the system. Thus, the *BD‐CTRP5* construct was used in Y2H screening against a universal, normalized human cDNA library (TaKaRa). A total of 56 positive clones were isolated from 4 × 10^6^ yeast transformants with a mating efficiency of 13%. After distinguishing genuine positive clones and eliminating duplicates, our high‐stringency plating conditions resulted in the identification of high temperature requirement A serine peptidase 1 (HTRA1) as one of the potential binding partners of CTRP5 (Figure 1a, b). The sequence of the clone encoded the carboxyl (C)‐terminal portion (aa 306–480) of HTRA1 that includes part of a serine protease domain and a complete PDZ [Post‐Synaptic Density protein 95 (PSD95), *Drosophila* Disc large (Dlg1), and Zonula Occludens‐1 (ZO‐1)] protein domain (Figure 1b).

The interaction of HTRA1 with S163R‐CTRP5 was compared with that of HTRA1 with WT‐CTRP5 (Figure 1c). Ten‐fold serial dilutions of yeast cells expressing the HTRA1 clone with either WT‐CTRP5 or S163R‐CTRP5 were grown on control plates (SD‐Leu‐Trp) and on selection plates additionally lacking histidine (SD‐Leu‐Trp‐His) with variable concentrations of 3‐aminotriazole (3‐AT) to score for growth. This analysis revealed similar binding affinities for WT‐CTRP5 and S163R‐CTRP5 to the proteolytically inactive, truncated form of HTRA1 (Figure 1c). Taken together, these results indicate that HTRA1 and CTRP5 interact with each other and that the S163R mutation in CTRP5 does not affect this interaction.

### 2.2 The interaction between CTRP5 and HTRA1 is confirmed by co‐immunoprecipitation (Co‐IP)

The interaction between CTRP5 and HTRA1 was evaluated by co‐immunoprecipitation (Co‐IP) using cell lysates of ARPE19 cells overexpressing WT‐CTRP5‐V5 or S163R‐CTRP5‐V5 fusion proteins. We observed that HTRA1 co‐immunoprecipitated with both WT‐CTRP5‐V5 and S163R‐CTRP5‐V5 (Figure 1d). A reverse co‐immunoprecipitation assay showed that WT‐CTRP5‐V5 and S163R‐CTRP5‐V5 co‐immunoprecipitated with HTRA1 (Figure 1e). Neither the WT‐CTRP5 nor the S163R‐CTRP5 showed any variation in their association with HTRA1. These studies confirm the interaction of HTRA1 with both WT‐CTRP5 and S163R‐CTRP5 observed using Y2H screening.

### 2.3 The PDZ‐ligand of CTRP5 recognizes HTRA1

**Figure 2:**
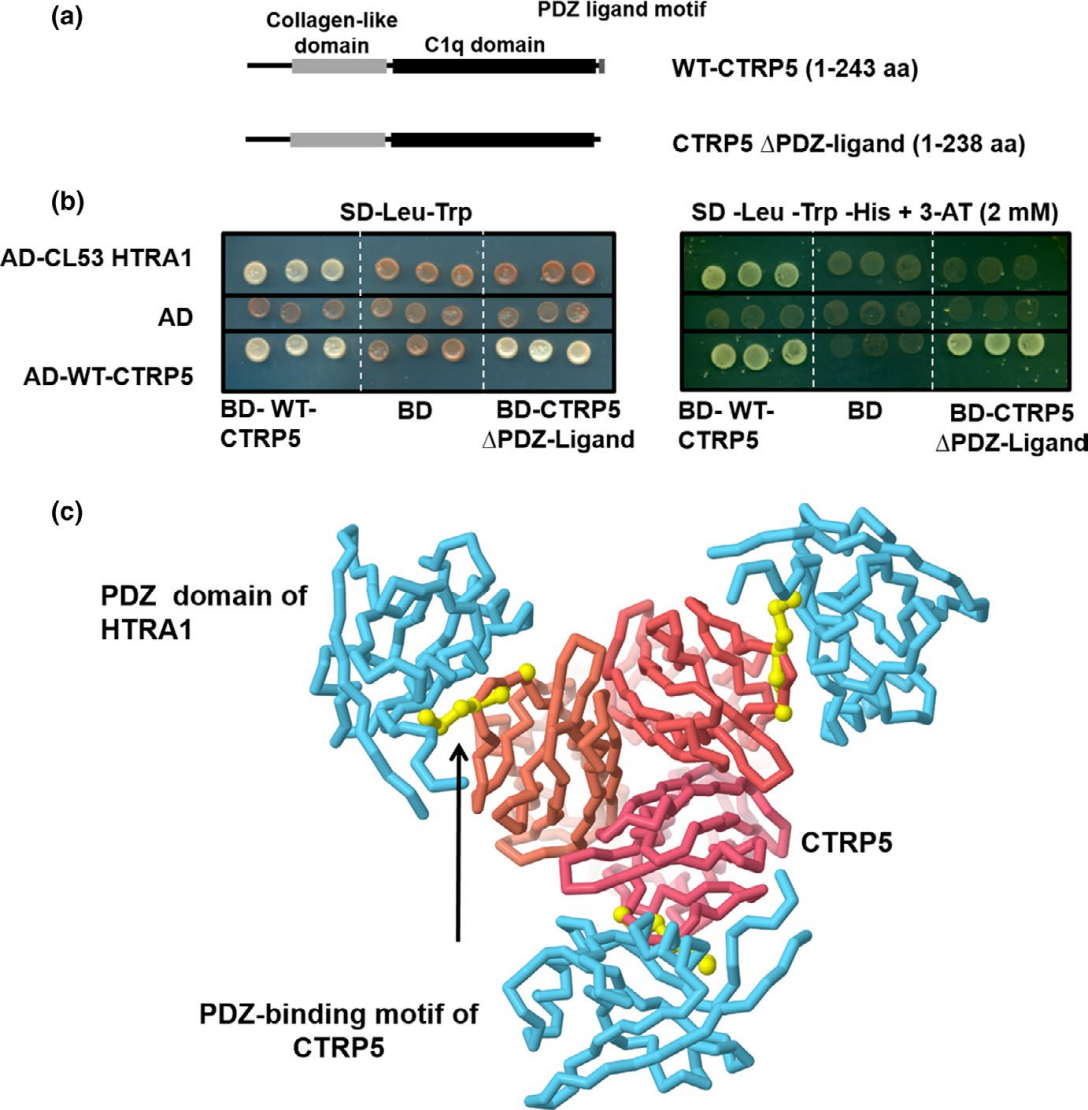
PDZ‐binding motif in CTRP5 mediates interaction between CTRP5 and HTRA1. (a) Schematic representation of full‐length CTRP5 and its truncated form lacking the C‐terminal PDZ‐ligand. These bait constructs were used to map the HTRA1 binding site in CTRP5 using the Y2H system. (b) Determination of HTRA1‐binding module in CTRP5. The indicated deletion construct of WT‐CTRP5 lacking its PDZ‐binding motif was fused with BD domain of GAL4 (BD‐CTRP5∆PDZ‐Ligand) and evaluated for its ability to interact with AD‐CL53 HTRA1. The interactions between the full‐length and truncated forms of WT‐CTRP5 were used as a positive control. (c) 3D reconstruction model indicating the interaction of WT‐CTRP5 and HTRA1 mediated by the PDZ domain in HTRA1. Protein data bank (PDB) entries of the trimeric CTRP5 (4f3j) and the PDZ domain of HTRA1 bound to a peptide (2joa) were overlapped to obtain a model of CTRP5‐HTRA1 interaction. CTRP5 is color‐coded in red, PDZ domain of HTRA1 is in blue, and the peptide from the PDZ‐binding motif of CTRP5 is in yellow

Since the sequence of the HTRA1 clone identified by Y2H contained a PDZ domain, a motif known to be critical for protein–protein interactions (Figure 1b), the CTRP5 sequence was analyzed for a PDZ‐binding motif using the Eukaryotic Linear Motif (ELM) resource (Dinkel et al., 2016). Such a PDZ‐binding motif was found at the C‐terminus of CTRP5 (Figure 2a). Moreover, in silico modeling of the interaction between WT‐CTRP5 and HTRA1 indicated a potential role of the PDZ‐domain of HTRA1 in its interaction with CTRP5 through its PDZ‐ligand (Figure 2c). However, based on this model, it is unclear whether trimeric or monomeric HTRA1 is involved in the interaction with CTRP5.

To experimentally validate the in silico findings, a C‐terminal truncation mutant, BD‐CTRP5 (∆PDZ‐ligand) (aa 1–238) (Figure 2a), was tested for its ability to bind HTRA1 using the Y2H HTRA1 clone. While the interaction of CTRP5 (∆PDZ‐ligand) with the full‐length WT‐CTRP5 was unaffected, deletion of the PDZ‐binding motif from WT‐CTRP5 significantly reduced its ability to interact with HTRA1 (Figure 2b). This result, along with the in silico modeling of the WT‐CTRP5/HTRA1 complex (Figure 2c), fits the model in which surface‐exposed, tri‐ or tetra‐peptide PDZ‐ligands, as seen in CTRP5, bind PDZ domains of HTRA proteases (Murwantoko et al., 2004).

### 2.4 CTRP5 enhances the elastase activity of HTRA1

**Figure 3:**
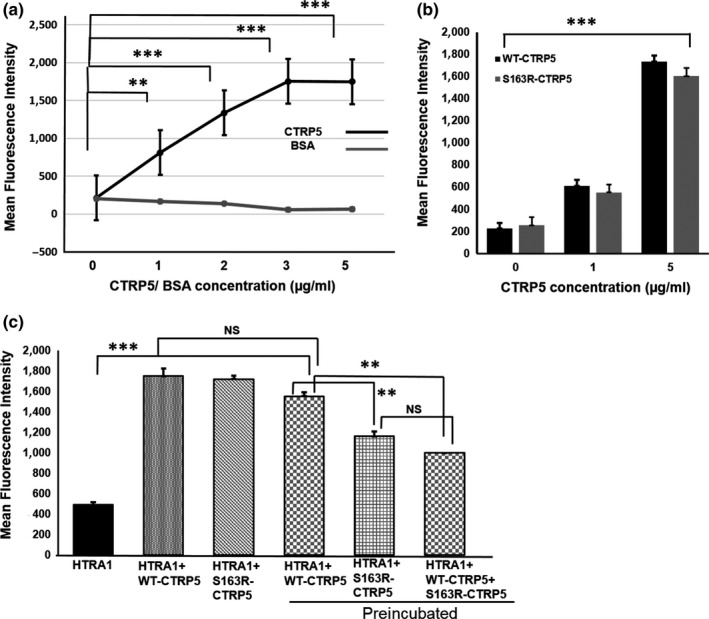
CTRP5 modulates elastase activity of HTRA1. (a) Different concentrations of bacterially expressed and purified full‐length WT‐CTRP5 (1–5 μg/ml) or BSA were incubated with 1 μg/ml HTRA1 for 60 min at room temperature. Elastin degradation was analyzed fluorometrically by measuring fluorescence intensity of cleaved BODIPYL‐elastin as a substrate in the reaction. BSA was used as a control protein to determine the specificity of activation of full‐length HTRA1 by WT‐CTRP5. Fluorescence intensities were normalized to background fluorescence emitted by BODIPYL‐elastin alone. Values were plotted in a line graph with different concentrations of CTRP5 or BSA. The differences in values of the mean fluorescence intensity at different CTRP5 concentrations, relative to that when no CTRP5 was added, were evaluated using Student's *t* test, and *p* values were calculated. The *p* values <.05, .01, and .001 are indicated with *, **, and ***, respectively. (b) Both purified WT‐CTRP5 and S163R‐CTRP5 at concentrations of 0, 1, and 5 μg/ml were incubated with 1 μg/ml of HTRA1 for 60 min at room temperature. Cleavage of BODIPYL‐elastin was measured fluorometrically and normalized to background fluorescence emitted by BODIPYL‐elastin alone and represented as a bar graph. *** denotes a *p* value of <.001. (c) Enhancement of HTRA1 protease activity by either WT‐CTRP5 or S163R‐CTRP5. Preincubation of WT‐CTRP5 or S163R‐CTRP5 for 60 min with HTRA1 prior to the addition of BODIPYL‐elastin substrate resulted in decreased elastase activity when compared to addition of WT‐CTRP5 or S163R‐CTRP5 to HTRA1 without preincubation. Preincubation of S163R‐CTRP5 with HTRA1 caused a significant decrease in HTRA1 activity when compared to preincubation of HTRA1 with WT‐CTRP5 (*p* < .01). Preincubation of an equimolar mixture of both WT‐CTRP5 and S163R‐CTRP5 with HTRA1 resulted in decreased HTRA1 activity when compared to preincubation of HTRA1 with WT‐CTRP5. Fluorescence intensities as a measure of activity of HTRA1 were graphically represented. The *p*‐values <.01 and.001 are indicated with ** and ***, respectively. NS indicates nonsignificance in the comparison of two data sets

To understand the potential impact of the interaction of CTRP5 with HTRA1, the protease activity of HTRA1 was tested in the presence of full‐length CTRP5. We performed an in vitro elastase degradation assay using DQ elastin, quenched by BODIPY‐FL dye, as a substrate to measure the activity of proteolytically active, recombinant HTRA1. While addition of BSA as a control had no effect on DQ elastin degradation, the presence of WT‐CTRP5 significantly increased the degradation of DQ elastin (Figure 3a; *p* < .002). Higher concentrations of WT‐CTRP5 (regardless of whether the CTRP5 used came from a commercial source or was purified in‐house) resulted in a linear increase in DQ elastin degradation (Figure 3a, b; Figure S2). Similarly, addition of S163R‐CTRP5 increased the elastase activity of HTRA1 (Figure 3b; *p* < .001).

Previous observations indicated that co‐expression of WT‐CTRP5 with S163R‐CTRP5 enhanced the formation of sub‐RPE deposits in mice (Dinculescu et al., 2015). Since, L‐ORD progresses with age, we tested whether preincubation of HTRA1 with WT‐CTRP5, S163R‐CTRP5, or a mixture of WT‐CTRP5 and S163R‐CTRP5 would affect the protease activity of HTRA1. Preincubation of WT‐CTRP5 with HTRA1 for 1 hr prior to the addition of DQ elastin enhanced the protease activity of HTRA1 compared with its activity in the absence of WT‐CTRP5 (*p < *.003). Preincubation with WT‐CTRP5 activated HTRA1 to almost similar levels as WT‐CTRP5 or S163R‐CTRP5 added to the reaction mixture without preincubation (Figure 3c). However, when S163R‐CTRP5 was preincubated with HTRA1, the protease activity of HTRA1 was significantly lower compared with the protease activity when preincubated with the WT‐CTRP5 (Figure 3c: *p* < .001) and was half the protease activity compared with the that measured without preincubation.

In order to mimic the dominant negative heterozygous condition resulting in the L‐ORD pathology in vivo, we incubated both WT‐CTRP5 and S163R‐CTRP5 in equal concentrations in vitro to observe the effect of this mixture on HTRA1 activity. Interestingly, preincubation with a mixture of WT‐CTRP5 and S163R‐CTRP5 in a 1:1 molar ratio also significantly reduced the elastase activity of HTRA1 when compared to its activity after preincubation with WT‐CTRP5 (Figure 3c; *p < *.042). Moreover, the activity of HTRA1 preincubated with the 1:1 mixture of WT‐CTRP5 and S163R‐CTRP5 was similar to the level of HTRA1 activity observed when preincubated with S163R‐CTRP5 alone (Figure 3c). These results indicate that WT‐CTRP5 enhances the elastase activity of HTRA1 with or without preincubation to similar extents. However, based on the above observations, we hypothesize that S163R‐CTRP5 only initially stimulates HTRA1 protease activity but subsequently reduces HTRA1 activity. In addition, when both the WT‐CTRP5 and S163R‐CTRP5 proteins are present in equimolar concentrations (i.e., conditions mimicking chronic exposure of these proteins to each other in vivo in a heterozygote), the presence of mutant S163R‐CTRP5 attenuates the activation of HTRA1 by WT‐CTRP5.

### 2.5 HTRA1 cleaves WT‐CTRP5, but not S163R‐CTRP5

The CTRP5 has been suggested to be a component of the extracellular matrix (ECM) (Tu & Palczewski, 2014). Because HTRA1 is a serine protease and is involved in ECM remodeling (An, Sen, Park, Gordish‐Dressman, & Hathout, 2010), we questioned whether WT‐CTRP5 and S163R‐CTRP5 were substrates of HTRA1 protease activity.

**Figure 4:**
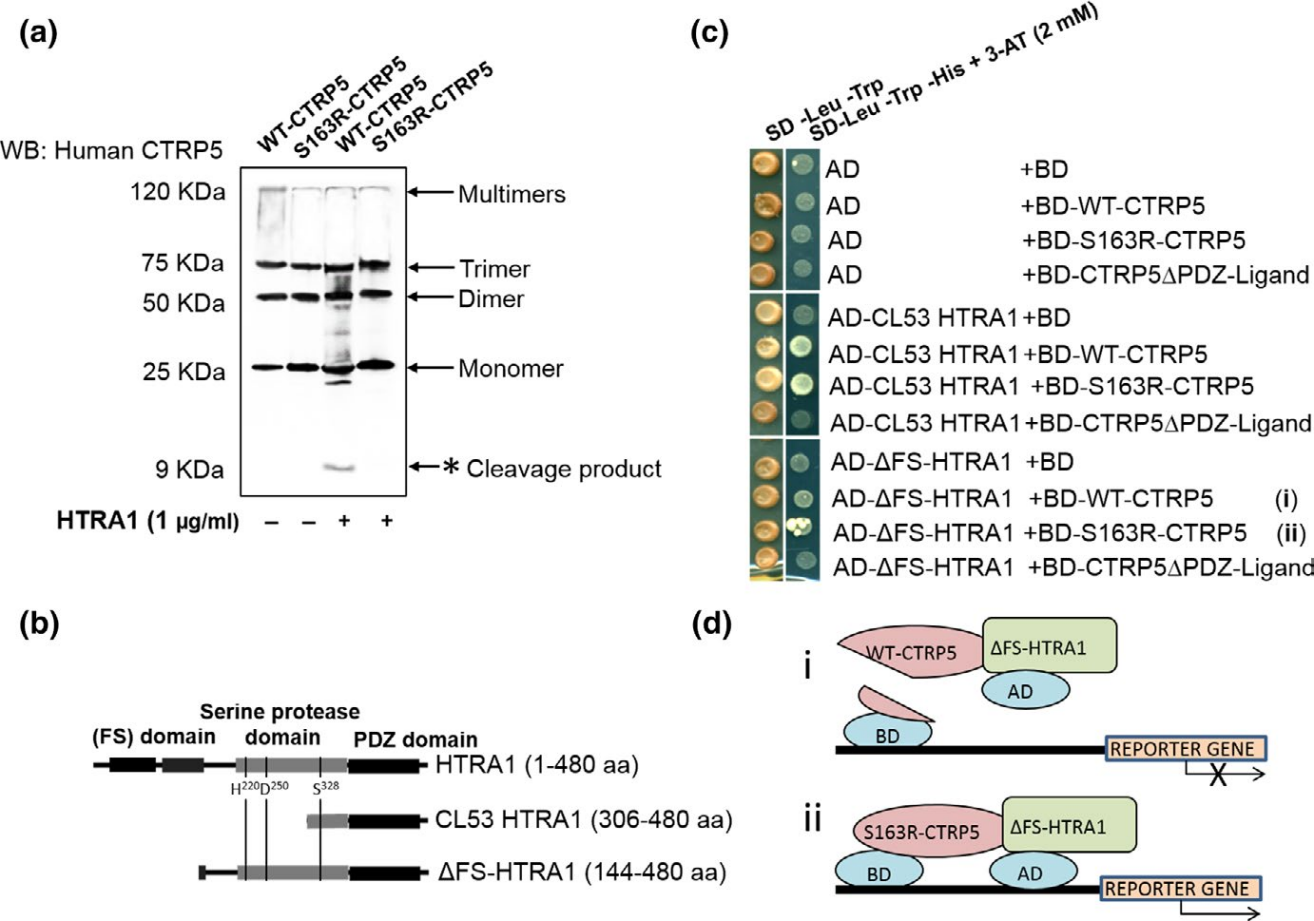
HTRA1 cleaves WT‐CTRP5 but not S163R‐CTRP5. (a) WT‐CTRP5 or S163R‐CTRP5 (5 μg/ml) was incubated at 37°C with HTRA1 (1 μg/ml) in a reaction buffer for 5 hr. The reaction mixture was resolved by SDS‐PAGE and subjected to immunoblotting with anti‐human CTRP5 antibody. Monomer, dimer, trimer, and multimers of the CTRP5 protein are indicated by arrows. A cleavage product at approximately 9 KDa when WT‐CTRP5, but not S163R‐CTRP5, is incubated with HTRA1 is shown with an asterisk. Incubations of WT‐CTRP5 or S163R‐CTRP5 alone without addition of HTRA1 were used as negative controls. (b) Schematic representation of the domain organization in truncated forms of HTRA1 prey constructs used in Y2H CTRP5 cleavage studies in comparison with full‐length HTRA1. The follistatin (FS) domain is a combination of the IGF‐binding protein (IGFBP) and the Kazal‐type serine protease inhibitor (KI) domains.The position of HTRA1 catalytic triad (Ser328, His220, and Asp250) was indicated. (c) Proteolytically active HTRA1 formed a stable complex with S163R‐CTRP5, but less efficiently with WT‐CTRP5. The truncated form of HTRA1 which has been previously reported as proteolytically active (Campioni et al., 2010) lacking its FS domain, but containing fully operational serine protease and PDZ domains (ΔFS‐HTRA1 (144–480 aa)) was fused with the AD domain of GAL4 and evaluated for its ability to form stable complex with WT‐CTRP5 and S163R‐CTRP5 mutant using the Y2H assay. To distinguish stable from transient protein–protein interactions, AD‐CL53 HTRA1 was used. Yeast cells carrying the proper combinations of bait and prey plasmids were plated on semi‐selective (SD‐Leu‐Trp) and selective (SD‐Leu‐Trp‐His) media supplemented with 3‐AT (2 mM). (d) A schematic of the Y2H assay depicting HTRA1‐CTRP5: (i) Schematic depicting interaction between BD‐ΔFS‐HTRA1 and BD‐WT‐CTRP5 in the Y2H assay. Binding of proteolytically active HTRA1 to WT‐CTRP5 results in the removal of the GAL4 DNA‐binding domain (BD) fused to the N‐terminal end of CTRP5 and affects reporter gene expression. (ii) Schematic depicting interaction between BD‐ΔFS‐HTRA1 and BD‐S163R‐CTRP5 that is unaffected due to S163R‐CTRP5 resistance to HTRA1 cleavage

An in vitro protease assay was performed by incubating proteolytically active recombinant HTRA1 with either WT‐CTRP5 or S163R‐CTRP5. This analysis revealed cleavage of WT‐CTRP5 resulting in the formation of at least one approximately 9 KDa cleavage product. Additional cleavage products might also have been present but were not immunoreactive to the polyclonal antibody used for detection. Similar analysis with S163R‐CTRP5 did not detect cleavage products of the mutant CTRP5 protein by HTRA1. These results suggest that WT‐CTRP5 serves as a substrate of HTRA1 and is cleaved by it, while S163R‐CTRP5 is resistant to HTRA1 cleavage (Figure 4a).

To confirm this result, we asked whether the Y2H interaction between HTRA1 and WT‐CTRP5 is abrogated by proteolytically active HTRA1. We generated HTRA1 protein (aa 144–480) (Figure 4b), previously reported to be proteolytically active (Campioni et al., 2010), and tested its ability to stably interact with WT‐CTRP5 or S163R‐CTRP5 in a Y2H assay. Cleavage of WT‐CTRP5 by proteolytically active HTRA1 would remove the GAL4 DNA‐binding domain (BD) fused to the N‐terminal end of CTRP5 and abolish the reconstitution of functional GAL4 transcription factor and activation of the downstream *HIS3* reporter gene (Figure 4b). Indeed, use of proteolytically active HTRA1 significantly reduced HTRA1/WT‐CTRP5 complex formation to undetectable levels. Binding of active HTRA1 to the mutant S163R‐CTRP5 was only moderately reduced when compared to the binding of proteolytically inactive HTRA1 to S163R‐CTRP5 (which could be explained by potential toxicity of active HTRA1 to the cells (Rigoulay, Poquet, Madsen, & Gruss, 2004)). The lack of GAL4 reconstitution in yeast cells containing protease‐active HTRA1 and WT‐CTRP5 (Figure 4d) supports the in vitro data that WT‐CTRP5 was cleaved by protease‐active HTRA1.

**Figure 5:**
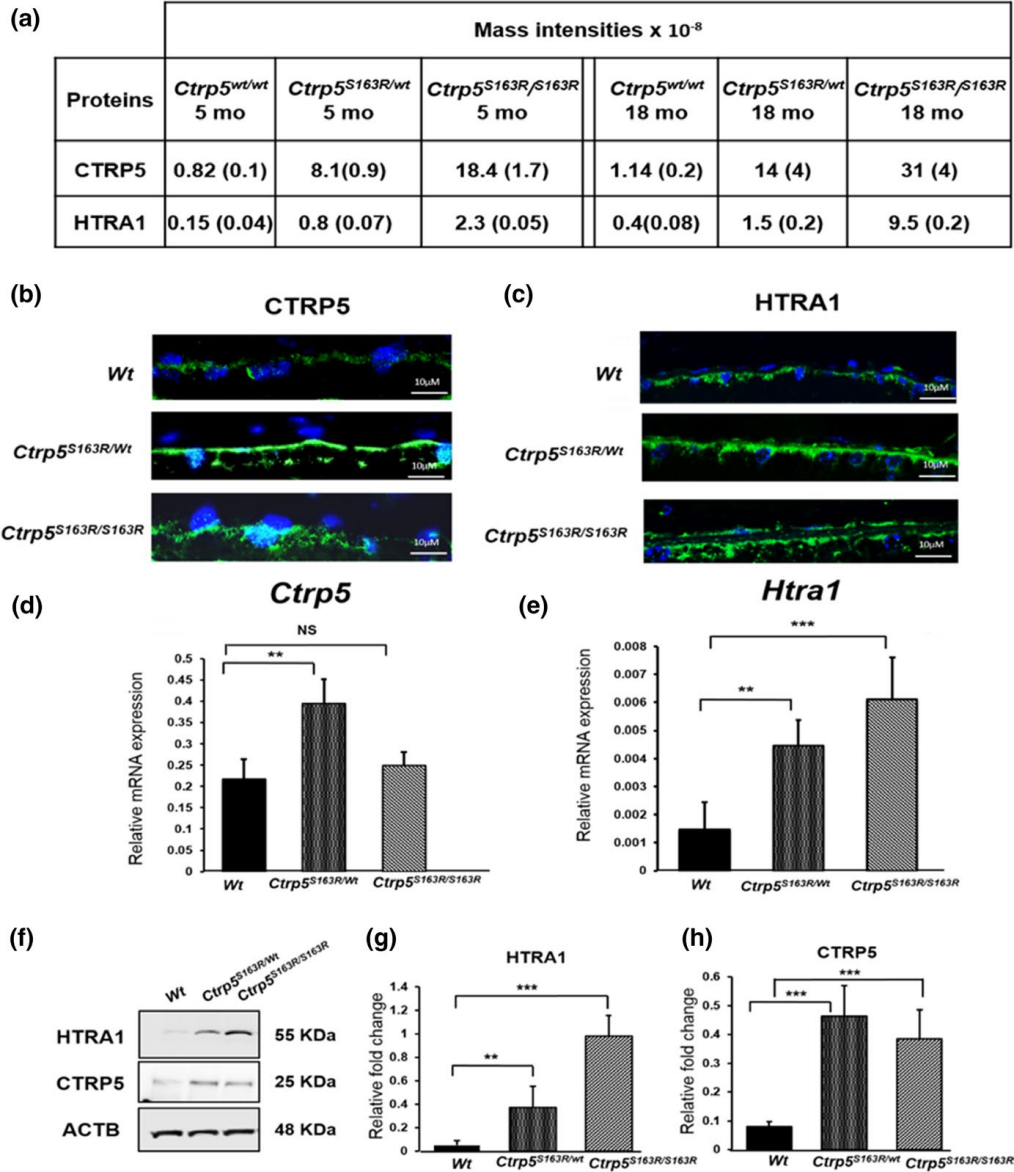
Accumulation of CTRP5 and HTRA1 in mice expressing mutant CTRP5. (a) Mass spectral analysis of BM‐Ch demonstrated increased levels of CTRP5 and HTRA1 proteins in *Ctrp5^S163R/wt^* and *Ctrp5^S163R/S163R^* mutant mice. Ages of mice were 5 months and 15–19 months (average 18 months). Mass intensities for CTRP5 or HTRA1 in the mutant mice and *Wt* mice at comparable ages were represented in the table. Two samples each from the mutant mice and four samples from *Wt* mice were analyzed. Each sample was a composite of BM‐Ch from four or more mice. The LFQ intensities from the samples for each genotype were averaged. Corresponding ± *SD* obtained were indicated in the parentheses. (b) Immunohistochemical analysis of the RPE layer from frozen retinal cryosections from 8‐ to 11‐month‐old *Ctrp5^S163R/wt^* and *Ctrp5^S163R/S163R^* mice (*n* = 3; one eye per mouse) and age‐matched *Wt* control mice (*n* = 3; one eye per mouse). Sections are stained with anti‐CTRP5 antibody to detect CTRP5 (Green). DAPI (Blue) was used to counterstain the RPE nuclei. (c) HTRA1 staining (Green) in *Ctrp5^S163R/wt^* and *Ctrp5^S163R/S163R^* (*n* = 3: one eye per mouse) and *Wt* control mice (*n* = 3: one eye per mouse) in the RPE layer from frozen retinal cryosections of 8‐ to 11‐month‐old Ctrp5S163R/wt and Ctrp5S163R/wt mice using anti‐HTRA1 antibody. DAPI (Blue) was used to counterstain the RPE nuclei. (d) Relative mRNA expression of *Ctrp5* to housekeeping gene *Gapdh* from posterior eyecups in 2‐ to 3‐month‐old *Ctrp5^S163R/wt^* and *Ctrp5^S163R/S163R^* mice (*n* = 8: one eye per mouse) compared to that in *Wt* mice (*n* = 8: one eye per mouse). Values were represented as mean (±*SD*). (e) Relative mRNA expression of *Htra1* to housekeeping gene *Gapdh* from posterior eyecups in 2‐ to 3‐month‐old mice with *Ctrp5^S163R/wt^* and *Ctrp5^S163R/S163R^* mice (*n* = 8: one eye per mouse) compared with that in *Wt* mice (*n* = 8: one eye per mouse). Values were represented as mean (±*SD*). (f) Immunoblot analysis from posterior eyecups of mice with various *Ctrp5* genotypes (*n* = 3) shown. Membranes were probed with anti‐CTRP5 and anti‐HTRA1 antibodies. Beta‐actin (ACTB) was used as a loading control. (g) Quantitation of levels of HTRA1 from immunoblot analysis. LICOR Image Studio Lite 5.2 was used for quantitation of Western blots. Levels of HTRA1 normalized to the levels of beta‐actin (ACTB) are represented graphically as relative fold change. The data obtained from immunoblot analysis were analyzed using Shapiro–Wilk test for normality and Student's *t* test to calculate the p values. The *p*‐values <.01 and.001 are indicated with ** and ***, respectively. NS indicates nonsignificance in the comparison of two data sets. (h) Quantitation of immunoblot detecting the levels of CTRP5 in mice posterior eyecup lysates normalized to the levels of beta‐actin

Taken together, these results establish that WT‐CTRP5, but not S163R‐CTRP5, is a substrate of HTRA1 and undergoes cleavage by HTRA1. The resistance of S163R‐CTRP5 to cleavage by HTRA1, together with its previously demonstrated tendency to aggregate, could explain the formation of thick deposits at the basal RPE (Figure 5b) and indicates a potential link between HTRA1 and CTRP5 pathology in the retina.

### 2.6 Mass spectrometry analysis of BM‐Ch from *Wt* and *Ctrp5* knock‐in mice reveals accumulation of CTRP5 and HTRA1 in basal deposits of L‐ORD mouse models

The formation of basal deposits associated with the BM is one of the key pathological features of both L‐ORD and AMD (Milam et al., 2000). Two mouse models of L‐ORD have been previously generated, involving either heterozygous (*Ctrp5^S163R/wt^*) (Chavali et al., 2011) or homozygous S163R mutations (*Ctrp5^S163R/S163R^*) (Borooah et al; unpublished data), respectively. Both models developed basal deposits associated with BM.

Using these two mouse models of L‐ORD, we examined the molecular mechanisms underlying L‐ORD pathology with a proteomic approach. Two samples from each type of mutant mice and 4 samples from *Wt* mice were analyzed. Each sample was a composite of BM‐Ch from four or more mice. Mass spectrometric analysis identified increased levels of both CTRP5 and HTRA1 in the BM‐Ch of both L‐ORD mouse models. Shown in Figure 5a are the levels of these proteins in *Ctrp5^S163R/wt^*, *Ctrp5^S163R/S163R^* and *Wt* mice. The LFQ (label‐free quantification) intensities for the proteins in each genotype were averaged. Levels of CTRP5 were 10‐fold higher in the BM‐Ch of *Ctrp5^S163R/wt^* mice than those in *Wt* mice as early as 5 months and increased to 12‐fold in 18‐month‐old mice (Figure 5a). In comparison, the levels of CTRP5 detected in the BM‐Ch tissue of 5‐month‐old and 18‐month‐old *Ctrp5^S163R/S163R^* mice were 22‐fold and 27‐fold higher, respectively, compared with the *Wt* mice (Figure 5a), suggesting an age‐dependent accumulation of CTRP5 in BM‐Ch.

The S163R mutation in CTRP5 resides in the tryptic peptide 162–170 (ASLQFDLVK). By comparing LFQ peptide intensities, it was determined that the intensity of this peptide in heterozygous BM‐Ch samples was approximately 40% of that found in wild‐type BM‐Ch samples. However, this peptide was not observed in *Ctrp5^S163R/S163R^* BM‐Ch samples (Table S2). The substitution of Arg for Ser at amino acid position 163 is predicted to introduce an additional tryptic cleavage site generating the shorter peptide, LQFDLVK. Following further analysis of the mass spectrometric data, the peptide sequence LQFDLVK was identified in all *Ctrp5^S163R/wt^* and *Ctrp5^S163R/S163R^* samples but not in *Wt* samples. Taken together, this indicates that both WT and S163R‐CTRP5 proteins were only present in BM‐Ch samples from *Ctrp5^S163R/wt^* mice, while only the mutant S163R‐CTRP5 peptide was present in *Ctrp5^S163R/S163R^* samples. Assuming that the mass spectral intensities were the same per unit WT and mutant peptide, about 2‐ to 3‐fold more mutant peptide than WT peptide was observed in the heterozygous samples. The 18‐month samples from *Ctrp5^S163R/S163R^* mice had about 3‐fold more mutant protein than the corresponding 18‐month samples from *Ctrp5^S163R/wt^* mice. Previous reports indicated that the expression of S163R‐CTRP5 leads to a reduction in the formation of WT‐CTRP5 octadecamers as a result of co‐aggregation of WT‐CTRP5 with S163R‐CTRP5 (Stanton et al., 2017). Accumulation of both WT‐CTRP5 and S163R‐CTRP5 in *Ctrp5^S163R/wt^* mice as well as stronger deposition of the S163R‐CTRP5 mutant in *Ctrp5^S163R/S163R^* mice supports these findings. Furthermore, the aggregation and age‐dependent progression of deposit formation caused by S163R‐CTRP5 observed in these studies mimic the phenotype seen in L‐ORD patients.

Mass spectrometric analyses also showed a significant accumulation of HTRA1 in the BM‐Ch basal deposits in both *Ctrp5^S163R/wt^* and *Ctrp5^S163R/S163R^* models (Figure 5a). The levels of HTRA1 in BM‐Ch tissue of *Ctrp5^S163R/wt^* mice were 5‐fold higher than that of *Wt* mice at 5 months and 3‐fold in 18‐month‐old mice. However, in *Ctrp5^S163R/S163R^* mice, the levels of HTRA1 were 15‐fold and 24‐fold higher in 5‐month‐old and 18‐month‐old animals, respectively, when compared with that in age‐matched *Wt* mice. These findings indicate the accumulation of HTRA1 in the BM‐Ch at the region of basal sub‐RPE deposits in both *Ctrp5^S163R/S163R^* and *Ctrp5^S163R/wt^* mouse models when compared to that of age‐matched *Wt* mice.

These results were confirmed by immunostaining of the retinal sections of *Ctrp5^S163R/wt^*, *Ctrp5^S163R/S163R^*, and *Wt* mice with antibodies specific to CTRP5 and HTRA1 which detected immunoreactive signals in all three genotypes in the basal RPE region. The corresponding hematoxylin‐and‐eosin‐stained retinal section indicated no gross abnormalities in the retinal architecture in *Ctrp5* mutant mice (Figure S5). However, the intensity of immunostaining for both CTRP5 and HTRA1 was much stronger in drusen‐like deposits in the sub‐RPE region of *Ctrp5^S163R/S163R^* and *Ctrp5^S163R/wt^* mice (Figure 5b, c and S4), further supporting the accumulation of these proteins in basal RPE deposits.

### 2.7 Expression of *Ctrp5* and *Htra1* in L‐ORD mouse models

We also asked whether the S163R mutation in the CTRP5 protein affected the regulation of *Ctrp5* and *Htra1* expression. Analysis of the expression of *Ctrp5* in posterior eyecup lysates by quantitative real‐time polymerase chain reaction (qRT‐PCR) showed that the levels of *Ctrp5* mRNA in *Ctrp5^S163R/wt^* mice were 2‐fold higher when compared to its expression in *Wt* mice (Figure 5d). However, the level of expression of *Ctrp5* in *Ctrp5^S163R/S163R^* mice was not significantly different from that observed in *Wt* mice (Figure 5d). In the case of *Htra1,* qRT‐PCR analysis of *Ctrp5^S163R/wt^* and *Ctrp5^S163R/S163R^* mice revealed a significant increase (3‐fold and 4‐fold, respectively) in the expression of *Htra1* when compared to that of *Wt* mice (Figure 5e; *p* < .003). However, the levels of CTRP5 detected by immunoblot analysis were similar in the posterior eyecup lysates of both *Ctrp5^S163R/wt^* and *Ctrp5^S163R/S163R^* mice aged 3–6 months, and higher compared to the levels observed in age‐matched *Wt* mice (Figure 5f). Consistent with these findings, increased levels of HTRA1 were observed by immunoblot analysis of these lysates (Figure 5f). Thus, while *Htra1* gene expression is correlated with HTRA1 accumulation in deposits and might contribute to it, *Ctrp5* gene expression does not reflect CTRP5 accumulation, at least in the basal deposits of *Ctrp5^S163R/S163R^* ^^mice.

### 2.8 Levels of HTRA1 substrates are elevated in L‐ORD mouse models

**Figure 6:**
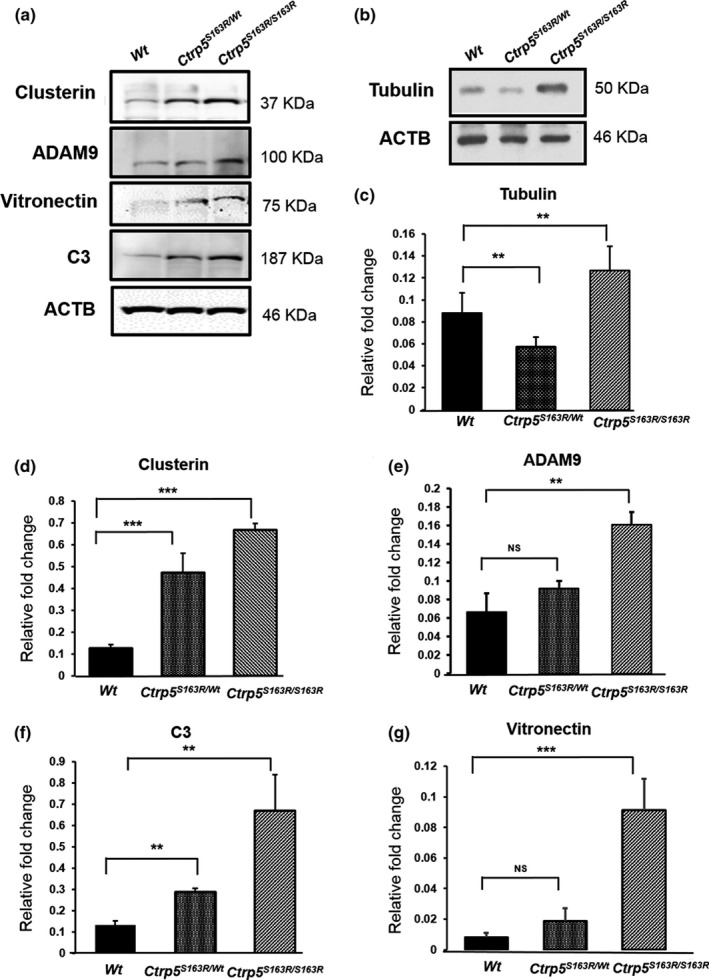
Analysis of proteins identified as HTRA1 substrates in both L‐ORD mouse models. (a) Immunoblot analysis of various HTRA1 substrates obtained from posterior eyecup lysates of 5‐ to 7‐month‐old mice from *Ctrp5^S163R/S163R^*, *Ctrp5^S163R/wt^*, and *Wt* mice. Blots were probed with clusterin, vitronectin, ADAM9, and C3 antibodies. Levels of these HTRA1 substrates were compared with ACTB protein as the loading control. (b) Levels of tubulin in posterior eyecup lysates of both *Ctrp5^S163R/S163R^* and *Ctrp5^S163R/wt^* mice, when compared to *Wt.* beta‐actin (ACTB), were used as loading control. Quantitation of levels of tubulin (c), clusterin (d), ADAM9 (e), C3 (f), and vitronectin (g) normalized to beta‐actin was represented as relative fold change. The data obtained from immunoblot analysis were analyzed using Shapiro–Wilk test for normality and Student's *t* test to calculate the *p* values. The *p*‐values <.01 and.001 are indicated with ** and ***, respectively. NS indicates nonsignificance in the comparison of two data sets

Recent studies show that a promoter variant causing increased expression of *HTRA1* in RPE cells leads to elevated levels of ECM proteins including HTRA1 substrates (Lin et al., 2018). Specifically, the HTRA1 substrates vitronectin, clusterin, Adam9, C3, and tubulin were also detected in the human RPE secretome (An et al., 2010; Melo et al., 2018). As the expression of *Htra1* is elevated in our L‐ORD mouse models, we investigated the impact of the increased *Htra1* on HTRA1 substrates in the RPE‐choroid of these mouse models using immunoblot analysis at the age of 3–6 months. Our studies showed increased levels of all HTRA1 substrates tested, except tubulin, in the lysates of posterior eyecups of both *Ctrp5^S163R/wt^* and *Ctrp5^S163R/S163R^* mice (Figure 6a). The levels of tubulin were higher in the posterior eyecup lysates of *Ctrp5^S163R/S163R^* 3‐ to 6‐month mice, when compared with *Wt*, but not in *Ctrp5^S163R/wt^* mice (Figure 6b). These results indicate that the S163R mutation in *Ctrp5* leads to the accumulation of HTRA1 substrates in the RPE‐choroid region of L‐ORD mouse models.

## 3 DISCUSSION

In the present study, we identified HTRA1 as a crucial player involved in the pathogenesis of L‐ORD. We observed that HTRA1 interacts strongly with CTRP5. In an independent proteomic screen focused on identifying proteins deposited in the sub‐RPE space, we found HTRA1 and CTRP5 as major proteins accumulated in *Ctrp5^S163R/wt^* and *Ctrp5^S163R/S163R^* L‐ORD mouse models when compared to *Wt* (Figure 5b and c). In addition, CTRP5 is observed to modulate the activity of HTRA1 and serves as a substrate of HTRA1 in vitro*.* These findings strongly suggest the involvement of HTRA1 in L‐ORD pathology.

Both CTRP5 and HTRA1 are components of the ECM and are expressed and secreted by RPE, the cell type that is primarily affected in L‐ORD. HTRA1 is an ubiquitous serine protease expressed in a wide variety of tissues (De Luca et al., 2003). It is suggested to play a crucial role in ECM reorganization and protein quality control during development and disease (Chamberland et al., 2009; Chien et al., 2004; Clausen, Kaiser, Huber, & Ehrmann, 2011; Grau et al., 2006; Nie et al., 2006; Tennstaedt et al., 2012). While CTRP5 is also expressed in multiple tissues, the physiological role of CTRP5 has not been established. Previous studies from our group and others have shown that WT‐CTRP5 forms multimers and that S163R‐CTRP5 enhances the formation of aggregates in cell culture models (Mandal, Vasireddy, Reddy, et al., 2006; Shu et al., 2011; Stanton et al., 2017) and in mice with adeno‐associated virus‐expressed S163R‐CTRP5 (Dinculescu et al., 2015). Consistent with these findings, we observed a significant accumulation of sub‐RPE deposits in the L‐ORD mouse models *Ctrp5^S163R/wt^* (Chavali et al., 2011) and *Ctrp5^S163R/S163R^* (Figure 5b), similar to the deposits reported in patients with L‐ORD (Milam et al., 2000). In addition, *Ctrp5^S163R/wt^* mice also exhibited dark adaptation abnormalities with significantly decreased electrophysiological responses. Progressive accumulation of autofluorescent spots along with compromised rod photoreceptor function was observed in this mouse model. Interestingly, detailed ultrastructural analysis of RPE of *Ctrp5^S163R/wt^* demonstrated the presence of vesicular structures filled with electron dense substance along with structural aberrations indicating RPE dysfunction (Chavali et al., 2011; Sahu et al., 2015). We have also observed formation of similar deposits in *Ctrp5^S163R/S163R^* mice (Borooah et al; unpublished data). (Chavali et al., 2011). Mass spectrometric analysis revealed the presence of both WT‐CTRP5 and S163R‐CTRP5 in *Ctrp5^S163R/wt^* (Figure 5a) and S163R‐CTRP5 in *Ctrp5^S163R/S163R^* mice. Accumulation of HTRA1 is also observed along with CTRP5 in sub‐RPE deposits of *Ctrp5^S163R/wt^* and *Ctrp5^S163R/S163R^* mice (Figure 5a). Taken together, these findings establish that S163R‐CTRP5, WT‐CTRP5, and HTRA1 contribute to the formation of sub‐RPE deposits observed in mouse models and patients with L‐ORD.

Deposit‐forming retinal phenotypes are observed in two monogenic, dominant macular degenerations, Doyne honeycomb retinal dystrophy (DHRD) due to mutations in EGF containing fibulin extracellular matrix protein 1 (EFEMP1) and Sorsby fundus dystrophy (SFD) due to mutations in tissue inhibitor of metalloproteinase‐3 (TIMP3), in addition to the common condition AMD (Fu et al., 2007; Klenotic, Munier, Marmorstein, & Anand‐Apte, 2004). HTRA1, which interacts with CTRP5, has been reported as a major protein associated with the AMD (Chong et al., 2015; Yan et al., 2018; Yang et al., 2006). All three diseases share phenotypic similarity with L‐ORD, including the formation of protein aggregates. Furthermore, CTRP5, EFEMP1, TIMP3, and HTRA1 implicated in these diseases are all ECM components (Jacobson et al., 2002; Marmorstein et al., 2002; Yang et al., 2006). This suggests a common mechanism underlying the pathology of these drusen‐forming retinal phenotypes.

Formation of high molecular weight protein aggregates has been suggested as the underlying cause of several neurodegenerative diseases including retinal degenerations (Dawson & Dawson, 2003; Illing, Rajan, Bence, & Kopito, 2002; Labbadia & Morimoto, 2013; Lee, Goedert, & Trojanowski, 2001; Selkoe, 2004; Tzekov, Stein, & Kaushal, 2011). Cellular stress due to accumulation of protein aggregates, and abnormal protein turnover has been implicated in the pathology of these conditions (Grune, Jung, Merker, & Davies, 2004; Grune, Reinheckel, Li, North, & Davies, 2002; Grune, Shringarpure, Sitte, & Davies, 2001). It is possible that the protein aggregates formed in the RPE of patients with the drusen‐deposit phenotype may exert cellular stress leading to RPE pathology (Usui et al., 2019). Alternatively, formation of the aggregates of ECM components CTRP5, HTRA1, EFEMP1, and TIMP3 may result in dysregulation of the extracellular environment of the RPE with high metabolic activity, leading to RPE abnormalities and deposit formation. Furthermore, it has been suggested that the accumulation of abnormal basal deposits in sub‐RPE with age may hinder the exchange of metabolites and nutrients between the RPE and choroid by forming a physical barrier resulting in age‐related degeneration of retinal tissue (Curcio & Millican, 1999; Kuntz et al., 1996; Spaide, Ooto, & Curcio, 2018). Any one of these or all three mechanisms may underlie the pathology of L‐ORD.

Our data from the proteolytic cleavage and Y2H assays demonstrate that WT‐CTRP5 is a substrate for HTRA1. However, the resistance of S163R‐CTRP5 to HTRA1 cleavage (Figure 4a) likely impacts its turnover and results in depletion of cleavage products and deposition of the protein. This resistance might be due to a conformational change in S163R‐CTRP5 that alters the surface properties and modulates both the cleavage efficiency of this protein as well as its aggregation tendency. Future studies focused on mapping the HTRA1 cleavage site in CTRP5, as well as understanding the role of CTRP5 cleavage products, may provide further insight.

ECM proteins such as Col3a1‐C are known to interact with HTRA1 through their PDZ‐ligands and enhance HTRA1 activity in vitro (Murwantoko et al., 2004). However Truebestein et al showed that PDZ domain of human HTRA1 is dispensable for its activation indicating the existence of alternative mechanisms of substrate‐induced HTRA1 activation (Truebestein et al., 2011). In the present study, in addition to serving as a substrate of HTRA1, CTRP5 also enhances the activity of HTRA1 by binding to the PDZ domain of HTRA1, suggesting a role for CTRP5 as an activator of HTRA1 and establishing physiological relevance to the interaction of these two proteins. Both proteins, WT‐CTRP5 and S163R‐CTRP5, contain PDZ‐binding motifs at their C‐termini, which can regulate the proteolytic activity of HTRA proteases. In solution, human HTRA1 exists primarily as a trimer regulated by an allosteric mechanism whereby activated monomers transfer the signal to other monomers of the trimer (Cabrera et al., 2017). Both the WT‐CTRP5 and S163R‐CTRP5 enhance the protease activity of HTRA1 in vitro (Figure 2b). However, we observed a reduction in HTRA1 activity in vitro, when HTRA1 was preincubated with S163R‐CTRP5 or a mixture of WT‐CTRP5 and S163R‐CTRP5 compared with WT‐CTRP5 alone (Figure 2c). If the in vitro preincubation assay conditions mimic the extracellular milieu of RPE with accumulated HTRA1 and S163R‐CTRP5, the HTRA1 activity in L‐ORD mouse models could be significantly lower compared with the *Wt* mice. The accumulation of HTRA1 substrates clusterin, vitronectin, C3, ADAM9, and tubulin detected in L‐ORD mouse models may indicate the lack of normal levels of HTRA1 activity and/or abnormal turnover of ECM proteins (Figure 6). Aberrant regulation of ECM has been well established in the pathology of macular degenerations with drusen‐deposit phenotype including AMD (Fernandez‐Godino, Pierce, & Garland, 2016; Khan et al., 2017, 2016). Similarly, dysregulation of the ECM in L‐ORD mouse models may play a role in disease pathology.

The S163R mutation in the *Ctrp5* gene is not only correlated with the accumulation of HTRA1, but also additionally with the elevated expression of the *Htra1* gene in L‐ORD mouse models (Figure 5e). Despite this increase in expression, accumulation of HTRA1 substrates noted in these mouse models is intriguing. It is likely that binding of HTRA1 with S163R‐CTRP5 may cause (a) aggregation of HTRA1 which physically separates HTRA1 and hinders access to its substrates and (b) steric hindrance for HTRA1 to interact with other substrates or to exert protease activity resulting in an accumulation of these substrates. While this requires further investigation, the accumulated HTRA1 substrates may result in a feedback regulatory signal leading to increased levels of *Htra1* expression similar to that observed in the L‐ORD mouse models.

Elevated HTRA1 expression is associated with AMD, arthritis, Alzheimer's disease, and Duchenne muscular dystrophy (Bakay, Zhao, Chen, & Hoffman, 2002; Gibbs et al., 2008; Hu et al., 1998; Tsuchiya et al., 2005; Yang et al., 2006). Specifically, the rs11200638 variant in the promoter region of *HTRA1,* which is identified as a major risk allele for AMD, results in elevated HTRA1 expression (DeWan et al., 2006). In addition, HTRA1 up‐regulation in RPE appears to result in RPE atrophy and photoreceptor degeneration as well as CNV (Jones et al., 2011; Kumar, Berriochoa, Ambati, & Fu, 2014; Melo et al., 2018). Although extrapolation of findings from in vitro studies suggests a potential reduction in the HTRA1 activity in the presence of the S‐163R‐CTRP5 in vivo, currently molecular assays to selectively measure HTRA1 activity in vivo in the L‐ORD mouse models have not been established. Future studies focused on HTRA1 activity in vivo may reveal the level of activity of HTRA1 and its potential role in RPE dysfunction and L‐ORD pathology.

In summary, our results suggest a complex interplay between HTRA1 and CTRP5 and point to a dual role of CTRP5 as an allosteric activator and a substrate for HTRA1. The present study shows that HTRA1/CTRP5 interaction has a physiological function and CTRP5, as an HTRA1 activator, may be involved in HTRA1‐dependent ECM remodeling. HTRA1 has been previously reported to cleave a wide variety of proteins which are involved in formation of sub‐retinal deposits and thereby functions in remodeling ECM within the sub‐retinal space. In this context, HTRA1 by its enzymatic activity may have the ability to influence protein turnover in the extracellular milieu of RPE cells. The drusen phenotype and the findings in the L‐ORD mouse models point to the dysfunction of ECM homeostasis as a central feature in the development of disease pathology. RPE plays a predominant role in age‐related changes in BM by formation of basal deposits either directly by altering expression and secretion of ECM proteins or indirectly by regulation of protein turnover in ECM (Bergen et al., 2018; Liu, Ye, Yanoff, & Li, 1997). The study of monogenic diseases like L‐ORD could provide a better understanding of the etiology of drusenogenesis as compared to studies of a more complex and multi‐factorial disease, such as AMD. Therefore, future studies aimed at analyzing the impact of other L‐ORD mutations identified recently on HTRA1 activity and the cleavage products of CTRP5 may shed light on the global mechanism of HTRA1‐mediated pathologies.

## 4 EXPERIMENTAL PROCEDURES

Detailed description of materials and methods used in the study was included in the Appendix S1.

## CONFLICT OF INTEREST

None declared.

## AUTHOR CONTRIBUTIONS

AC and KZR: designed and performed the majority of experiments and prepared the manuscript. AC: coordinated the study with collaborators, maintained mouse models, provided tissue samples for analysis, and prepared purified proteins. DG: performed proteome analysis and analyzed data. ASH, VK, MV, PB; performed experiments. AC, SB, DG: *involved in *in silico structure modeling. SB: *involved in* data interpretation and manuscript writing. CS, CH: provided CTRP5 plasmids, interpreted the data, and wrote the manuscript. PS: provided HTRA1 plasmid and interpreted the data. RA, SS: designed, supervised the study, interpreted the data, prepared the manuscript, and acquired funding. RA: developed the concept of the study.
