# Supplementary figures and images for: Late‐onset retinal degeneration pathology due to mutations in CTRP5 is mediated through HTRA1
Source: Aging Cell. 2019 Aug 5;18(6):e13011. doi: 10.1111/acel.13011 (PMC6826137; doi:10.1111/acel.13011)

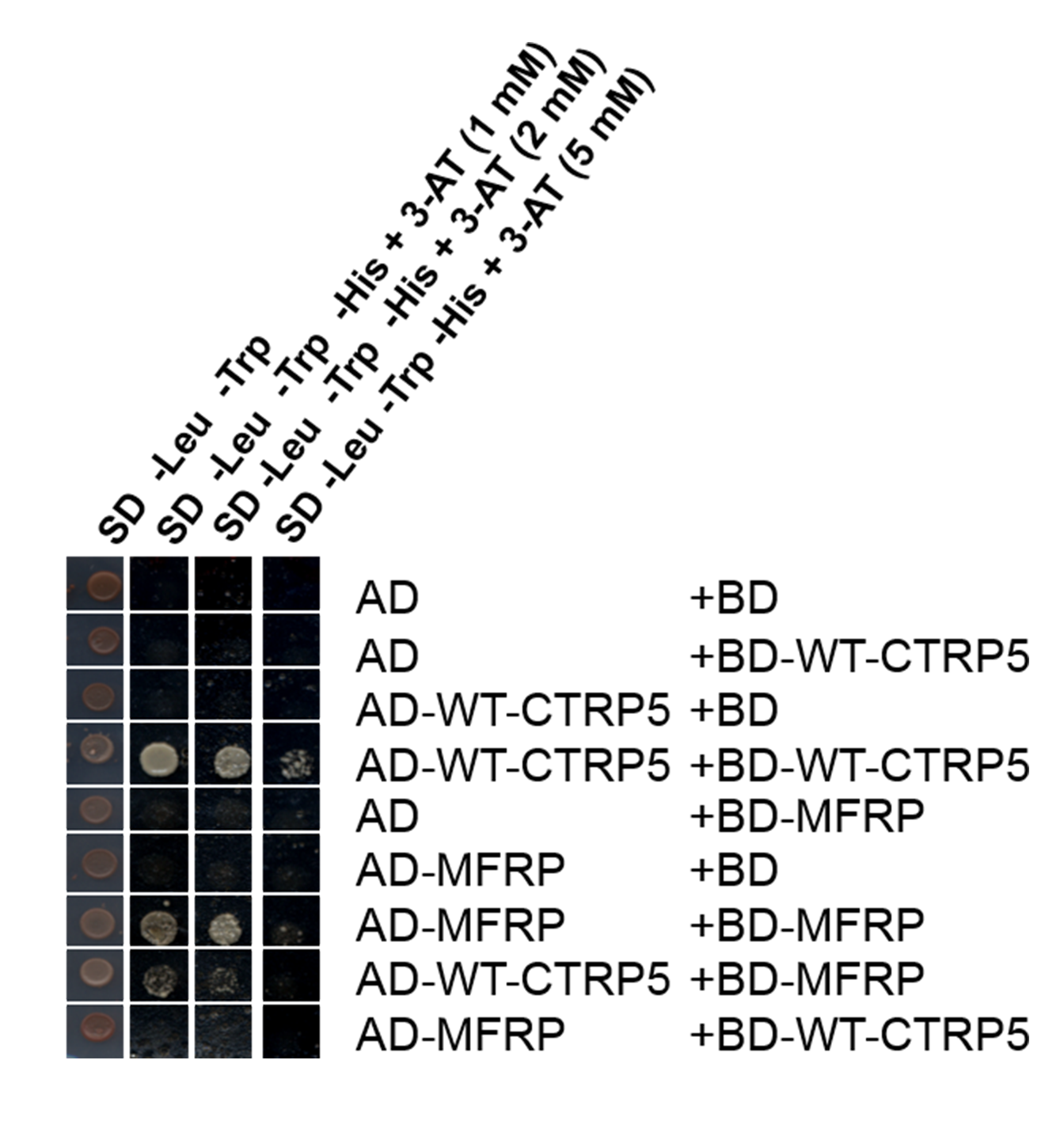

Supplement: Supplementary file 1 [file ACEL-18-e13011-s001.tif]

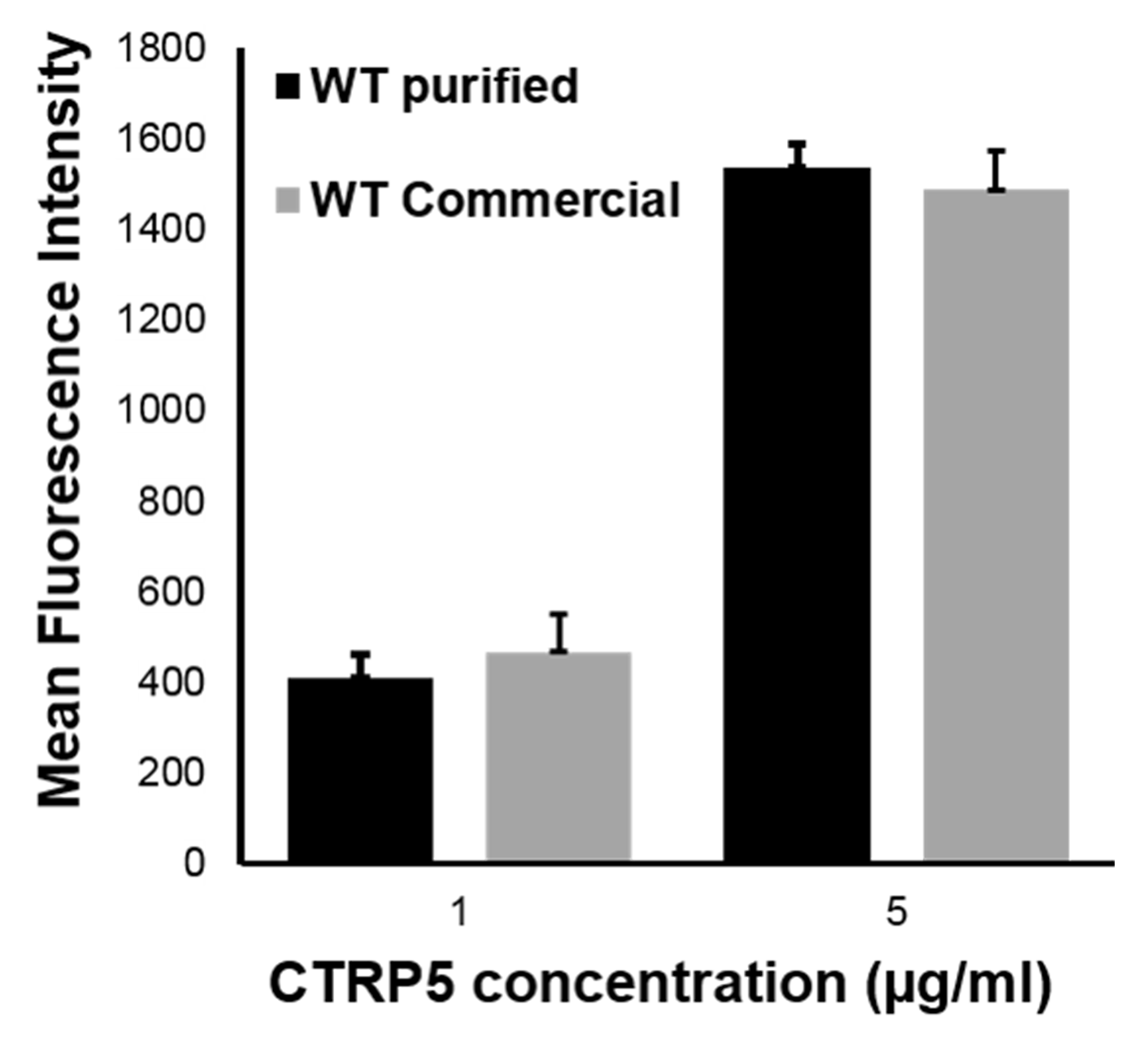

Supplement: Supplementary file 2 [file ACEL-18-e13011-s002.tif]

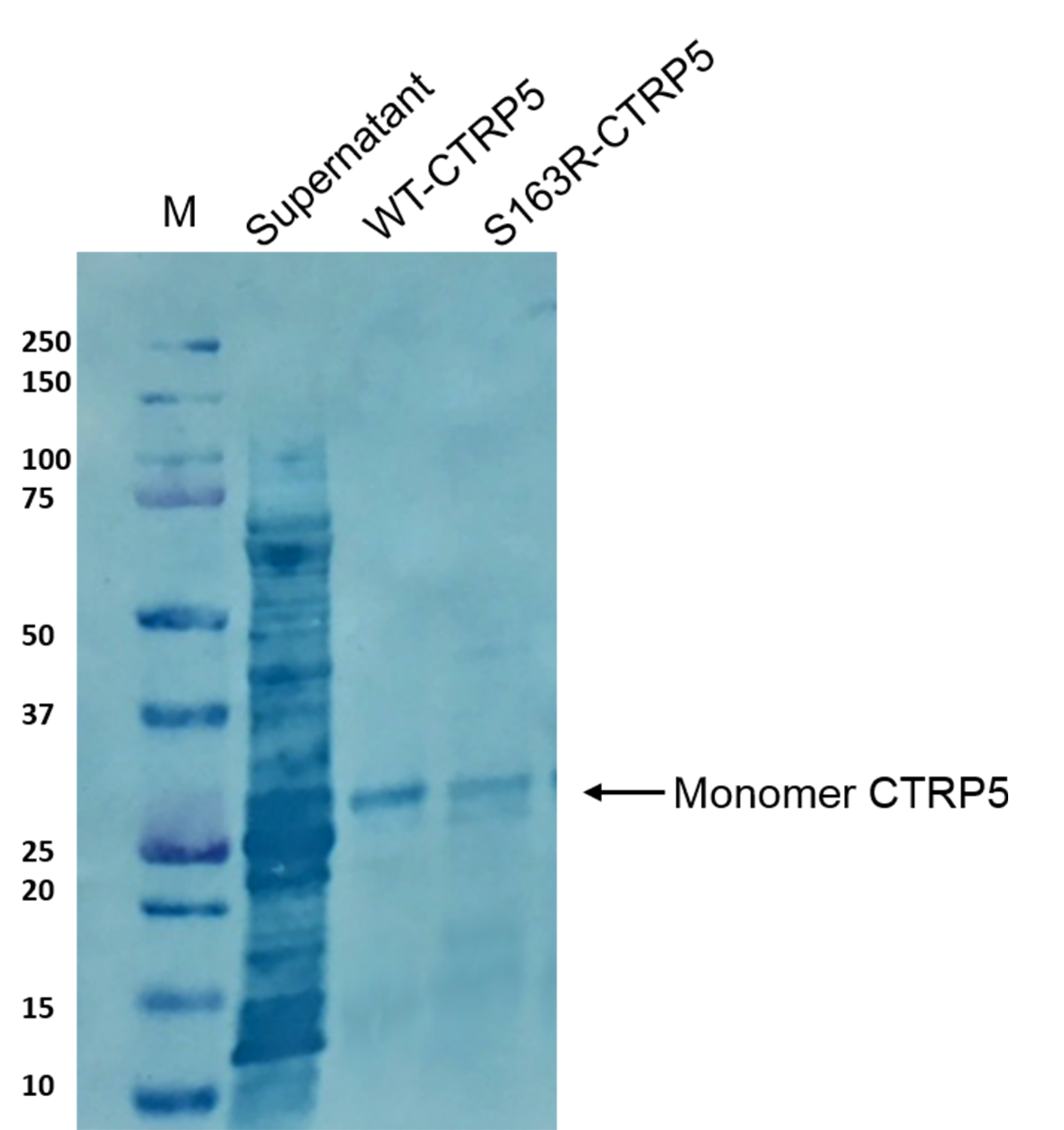

Supplement: Supplementary file 3 [file ACEL-18-e13011-s003.tif]

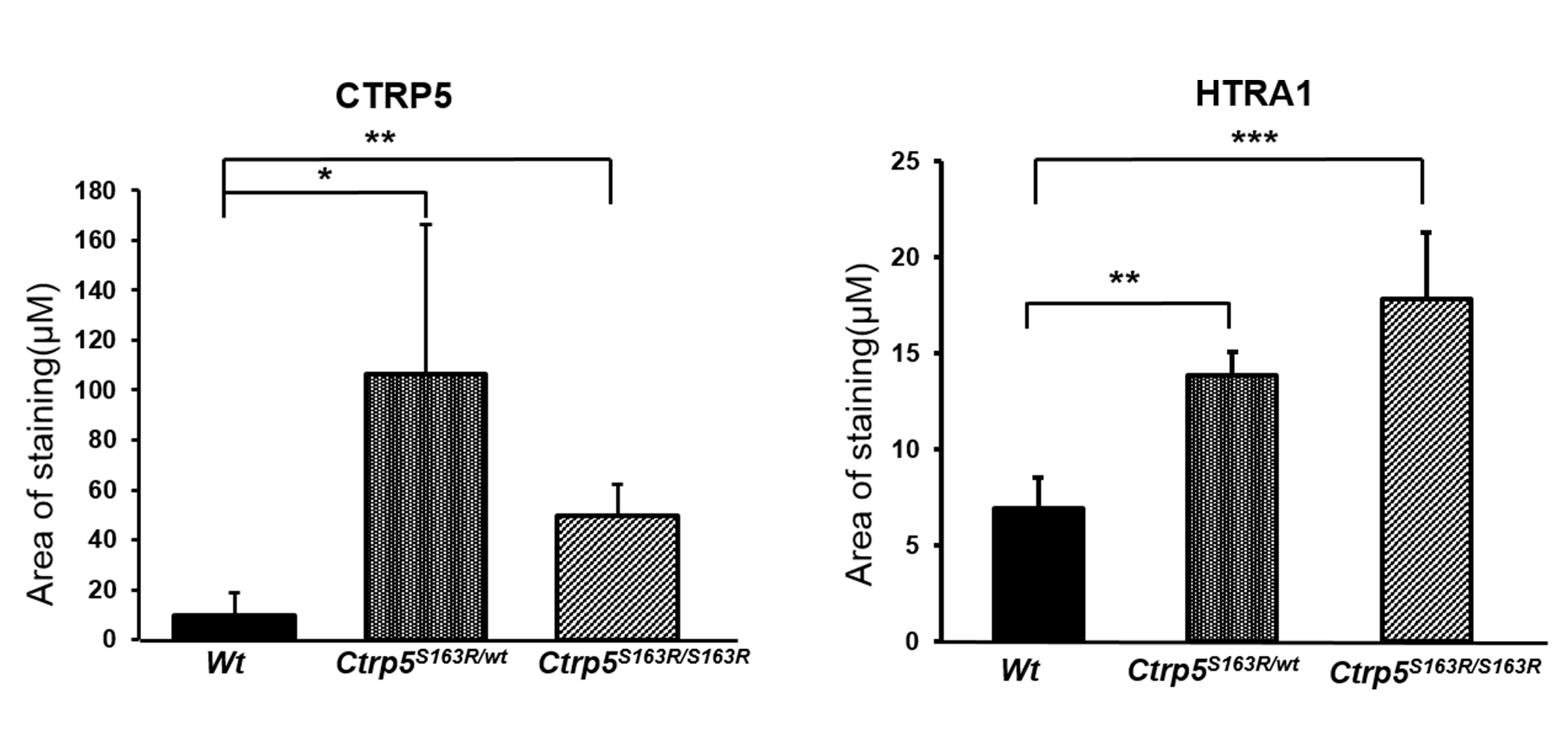

Supplement: Supplementary file 4 [file ACEL-18-e13011-s004.tif]

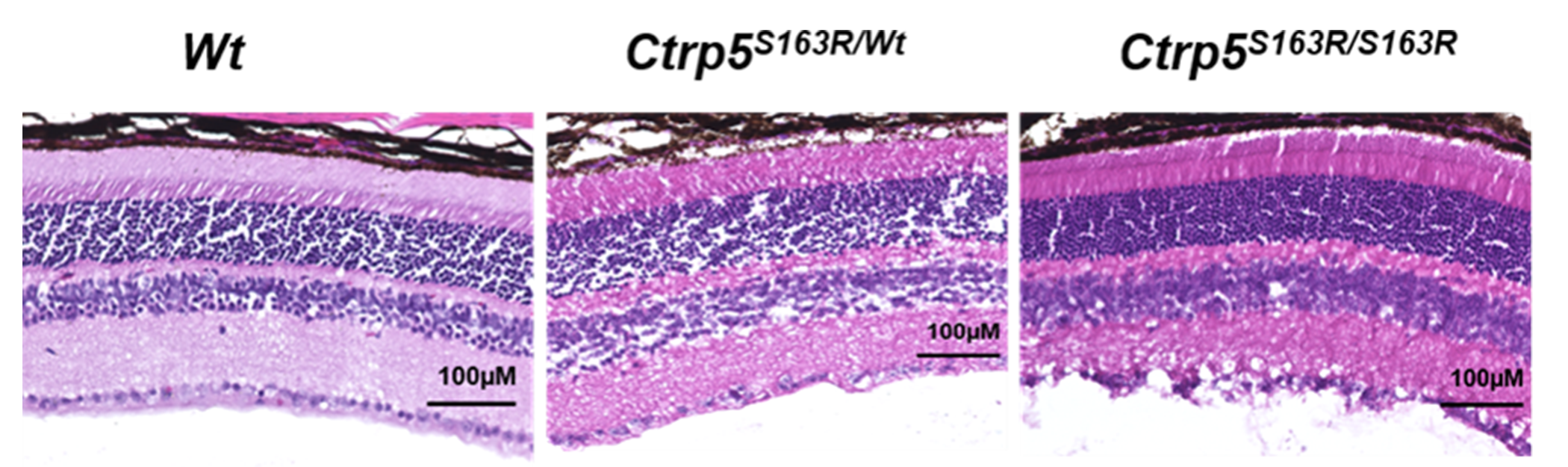

Supplement: Supplementary file 5 [file ACEL-18-e13011-s005.tif]
