# Supplementary material for: Late‐onset retinal degeneration pathology due to mutations in CTRP5 is mediated through HTRA1
Source: Aging Cell. 2019 Aug 5;18(6):e13011. doi: 10.1111/acel.13011 (PMC6826137; doi:10.1111/acel.13011)
Supplement: Supplementary file 6 [file ACEL-18-e13011-s006.docx]

**Table S1**

| ***Saccharomyces cerevisiae* strains** | | | | | |
| --- | --- | --- | --- | --- | --- |
| **Strain** | **Genotype** | | | | **Reference** |
| AH109 | *MATa, trp1-901, leu2-3, 112, ura3-52, his3-200, gal4Δ, gal80Δ, LYS2∷GAL1_UAS_-GAL1_TATA_-HIS3, GAL2_UAS_-GAL2_TATA_-ADE2, URA3∷MEL1_UAS_-MEL1_TATA_-lacZ* | | | | TaKaRa |
| Y2HGold | *MATa, trp1-901, leu2-3, 112, ura3-52, his3-200, gal4Δ, gal80Δ, LYS2::GAL1_UAS_-GAL1_TATA_-HIS3, GAL2_UAS_-Gal2_TATA_-ADE2 URA3::MEL1_UAS_-MEL1_TATA_ AUR1-C MEL1* | | | | TaKaRa |
| **Plasmids for Y2H screening** | | | | | |
| **Plasmid name** | **AD-fusion protein** | **BD-fusion protein** | **Background** | **Selectable marker** | **Reference** |
| pKZR042 | WT-CTRP5 (1-243 aa) |  | pDEST-AD | Amp/Leu | this study |
| pKZR043 |  | WT-CTRP5 (1-243 aa) | pDEST-BD | Kan/Trp | this study |
| pKZR044 |  | WT-CTRP5 (1-243 aa) | pGBKT7 | Kan/Trp | this study |
| pKZR045 |  | S163R-CTRP5 (1-243 aa) | pGBKT7 | Kan/Trp | this study |
| pKZR046 | CL53 HTRA1 (306-480 aa) |  | pGADT7-RecAB | Amp/Leu | TaKaRa (Universal Human Normalized Mate & Plate™ Library) |
| pKZR047 |  | WT-CTRP5 ∆PDZ-Ligand (1-238 aa) | pGBKT7 | Kan/Trp | this study |
| pKZR048 |  | MFRP | pDEST-BD | Kan/Trp | this study |
| pKZR049 | MFRP |  | pDEST-AD | Amp/Leu | this study |
| pKZR050 | ΔFS-HTRA1 (144-480 aa) |  | pGADT7 | Amp/Leu | this study |
| **Plasmids for protein expression in *E.coli*** | | | | | |
| **Plasmid name** | **Protein** | | **Back ground** | **Selectable marker** | **Reference** |
| pDEST14-C1QTNF5 | WT-CTRP5-His | | pDEST14 | Amp | Stanton et al 2017 |
| pDEST14-C1QTNF5-S163R | S163R-CTRP5-His | | pDEST14 | Amp | Stanton et al 2017 |
| **Plasmids for expression in mammalian cell lines** | | | | | |
| **Plasmid name** | **Protein** | | **Back ground** | **Selectable marker** | **Reference** |
| pcDNA 3.1-CTRP5-V5 | WT-CTRP5-V5 | | pcDNA 3.1/V5-His A | Amp | this study |
| pcDNA 3.1-CTRP5-S163R-V5 | S163R-CTRP5-V5 | | pcDNA 3.1/V5-His A | Amp | this study |
