# Supplementary material for: Late‐onset retinal degeneration pathology due to mutations in CTRP5 is mediated through HTRA1
Source: Aging Cell. 2019 Aug 5;18(6):e13011. doi: 10.1111/acel.13011 (PMC6826137; doi:10.1111/acel.13011)
Supplement: Supplementary file 7 [file ACEL-18-e13011-s007.docx]

| **Mass intensities x 10^-8^** | | | | | | | |
| --- | --- | --- | --- | --- | --- | --- | --- |
| **Peptide** | **Sequence** | **Ctrp5^Wt/Wt^**  **5 mo** | **Ctrp5^S163R/Wt^**  **5 mo** | **Ctrp5^S163R^/^S163R^**  **5 mo** | **Ctrp5^Wt/Wt^**  **18 mo** | **Ctrp5^S163R/Wt^**  **18 mo** | **Ctrp5^S163R^/^S163R^**  **18mo** |
| **128-142** | **VLLNEQGHYDPTTGK** | **1.3** | **4.9** | **8.2** | **1.4** | **10.0** | **21.0** |
| **162-170** | **ASLQFDLVK** | **0.5** | **0.6** | **0** | **0.4** | **1.2** | **0** |
| **164-170** | **LQFDLVK** | **0.1** | **1.8** | **2.1** | **0** | **2.1** | **6.5** |
