# Supplementary material for: Late‐onset retinal degeneration pathology due to mutations in CTRP5 is mediated through HTRA1
Source: Aging Cell. 2019 Aug 5;18(6):e13011. doi: 10.1111/acel.13011 (PMC6826137; doi:10.1111/acel.13011)
Supplement: Supplementary file 8 [file ACEL-18-e13011-s008.docx]

**Supplementary information:**

**Materials and methods**

**Animal usage**

Maintenance and experimental procedures on mice were performed in accordance to protocols approved by the UCSD INSTITUTIONAL ANIMAL CARE AND USE COMMITTE. The *Ctrp5^S163R/wt^* mice were generated in this laboratory and previously described (Chavali et al. 2011); *Ctrp5^S163R/S163R^* mice were also generated in this laboratory (Borooah et al. unpublished data).

**Plasmid construction**

Plasmids used for these studies were verified by DNA sequencing and/or by restriction digestion and are described in Table S1. Details regarding their construction are available upon request. For pDEST plasmid construction, cDNA cloning into pENTR™/D-TOPO vector and Gateway LR recombination reactions were done as described in the Gateway® Technology (Invitrogen, 12535-019 and 12535-027).

**Library screening and protein interaction tests in Y2H system**

For Y2H analysis, the GAL4-based Matchmaker Y2H system (TaKaRa) was utilized. Full-length open reading frames or truncated forms were inserted in pDEST-GADT7 (*Arabidopsis* Biological Resource Center, CD3-763; Shaw Laboratory) (AD), pDEST-GBKT7 (*Arabidopsis* Biological Resource Center, CD3-764; Shaw Laboratory) and pGBKT7 (TaKaRa, 630443), (BD) plasmids. The *S. cerevisiae* strains AH109 and Y2H gold were used (Table S1). For library screening, the Mate & Plate™ Universal Human Normalized Library was used and the transformation of yeast cells was performed according to the protocol for the Matchmaker™ Gold Y2H System (<https://www.takarabio.com)>.

Approximately 4 x 10^6^ transformants with achieved mating efficiency of 13% were grown on synthetic dropout (SD) medium (-Leu, -Trp, -Ade) (Sunrise Science; 1025) to select positive clones. Plates were incubated at 30° C and results were analyzed after 3-7 d. Positive interactions of selected clones were confirmed by testing their ability to activate other reporter genes, *HIS3* and *MEL1*. Subsequently, “prey” plasmids from selected clones were rescued and analyzed by sequencing. True positive prey clones were verified by retransformation and analysis of their ability to activate the reporter genes in the presence of the “bait”.

For small scale transformation, the Li/Ac-single-stranded carrier, DNA-PEG method (Gietz and Woods 2002) was used following the “Quick and Easy TRAFO Protocol” available at <https://home.cc.umanitoba.ca/≫gietz/Quick.html> which allows for simultaneous transformation of yeast with defined “bait” and “prey” plasmids. After transformation, yeast was grown on synthetic dropout (SD) medium (-Leu, -Trp) (Sunrise Science; 1012) to select for transformants containing the introduced plasmids. Plates were incubated at 30° C for up to 5 d. Next, three representative transformants from each strain were grown on proper selective media: SD medium (-Leu, -Trp), SD medium (-His, -Leu, -Trp) (Sunrise Science; 1014) w/ or w/o 3-amino-1,2,4-triazole (3AT, Sigma-Aldrich’ A8056) at the concentration indicated in the figure and/or SD medium (-Leu, -Trp, -Ade) to test protein interactions. Results were analyzed after 3-7 days.

**Cell culture:** ARPE 19 cells were obtained from ATCC. Cells were partially differentiated by growing them in DMEM media with high glucose (4.5g/L) and pyruvate supplemented with 3% heat inactivated fetal calf serum. All reagents were obtained from Thermo Fisher Scientific unless indicated. A final concentration of 1% Penicillin-streptomycin solution containing 10,000 U penicillin and 10 mg streptomycin was added to the growth media. Cells were maintained in humidified incubator at 37^0^C and 5% C02. ARPE19 cells from passages P10-P22 were used for the study.

**Co-immunoprecipitation assay**

ARPE-19 cells were transfected with WT-CTRP5-V5 or S163R-CTRP5-V5 constructs (Table S1) using Lipofectamine 3000^TM^ according to manufacturer’s instructions (Thermo Fisher Scientific; L3000008). Forty-eight hours after transfection, cells were harvested and lysed in immunoprecipitation buffer containing 50 mM HEPES, pH 7.4, 150 mM NaCl, 1% NP-40, 0.5% sodium deoxycholate and protease inhibitors. Cells were kept on ice for 20 min for complete lysis and centrifuged at 13000 rpm for 15 min at 4^0^ C to remove cell debris as well as unlysed cells. The supernatant was used for co-immunoprecipitation experiments using a Pierce co-immunoprecipitation kit according to manufacturer’s instructions (Thermo Fisher Scientific; 26149). Anti-V5 tag and HTRA1 rabbit polyclonal antibodies were used for immunoprecipitation and normal rabbit IgG was used as a negative control. Ten percent of total reaction served as the input. Immunoprecipitates were analyzed by SDS-PAGE and HTRA1 and CTRP5 were detected using HTRA1 and anti V5-Tag antibodies, respectively.

**Protein purification**

BL 21 star (DE3) bacterial cells expressing either WT-CTRP5 or S163R-CTRP5 were grown in LB media at 22^0^C for 6 hours, followed by 20 hours induction with 0.5mM Isopropyl β-D-1-thiogalactopyranoside (IPTG). Cells were recovered by centrifugation, then resuspended in 200 ml 10 mM Tris, pH.8 per 2 liter cells and frozen overnight at -80^0^C. Cells were lysed by freeze-thawing, followed by treatment in a Waring blender for 30 sec to help shear DNA. Cell lysates were centrifuged at 10000g for 2 hours.  Supernatants were decanted and filtered through 0.45 micron filter. Cell pellets were resuspended in the buffer containing 200 ml 5M GuHCl, pH 7.5 and agitated for 1hour; the supernatants were decanted and filtered as above. Supernatants were passed through nickel sepharose and washed with PBS, followed by 25 mM imidazole in PBS until protein concentration reached the back ground levels. (Approximately 200 ml each). Nickel bound proteins were eluted in PBS plus 500 mM imidazole. Peak fractions were concentrated and washed 3x with PBS using a 10 KDa cutoff centrifuge concentrator. The eluate was dialyzed against buffer containing 50 mM formic acid overnight, diluted 3x with 10 mM sodium phosphate, pH 6, dialyzed for 4 hours in the buffer containing 10 mM sodium phosphate, pH 5.6, 0.2% beta mercaptoethanol, concentrated and aliquoted. Protein estimation was performed using Bradford assay and purity of the fractions was analyzed by using SDS-PAGE and western blot analysis (Figure S3).

**Elastase activity assay**

Elastase activity assay of HTRA1 was performed using the ENcheck elastase activity assay kit (Thermo Fisher Scientific; E12056) according to manufacturer’s instructions. This assay is based on *in vitro* degradation of elastin by recombinant HTRA1 using DQ elastin, a soluble elastin labeled with quenched BODIPY FL dye, as a substrate. Indicated concentrations of full length recombinant WT-CTRP5 and S163R-CTRP5 were added to the *in vitro* reaction with DQ elastin and HTRA1 and incubated at 37^0^ C for 1 hour. Degradation of elastin products by HTRA1 with or without CTRP5 were analyzed by fluorimetry. Porcine elastase was used as positive control in the reaction and bovine serum albumin (BSA) was used as negative control. Recombinant human WT-CTRP5 and S163R-CTRP5 proteins were obtained by bacterial expression of codon optimized constructs and purified as explained previously and described above in detail (Stanton et al. 2017)

**CTRP5 cleavage assay**

Degradation of CTRP5 by proteolytic active HTRA1 was analyzed using recombinant HTRA1 (Thermo Fisher Scientific; RP 77538). Recombinant HTRA1 1 µg/ml was incubated with WT-CTRP5 or S163R-CTRP5 at a final concentration of 5 µg/ml in a reaction buffer containing 50 mM Tris-HCl, pH7.5, 150 mM NaCl. The reaction mixture was incubated at 37^0^ C for 5 hours and analyzed by immunoblot. Degradation of CTRP5 was analyzed by resolving the reaction mixture on a 12% gel and followed by immunoblot. The extent of CTRP5 cleavage was analyzed by probing the membranes using rabbit anti-human polyclonal CTRP5 antibody.

**Immunoblot analysis**

Cells were lysed in RIPA buffer containing 20 mM Tris-HCl, pH 7.5, 150 mM NaCl, 1 mM Na_2_-EDTA, 1 mM EGTA, 1% NP-40, 1% sodium deoxycholate, 2.5 mM sodium pyrophosphate, 1 mM β-glycerophosphate, 1 mM Na_3_VO_4_ and a protease inhibitor cocktail, followed by incubation on ice for 20 min. After centrifugation at 13000 rpm at 4^0^ C for 15 min, the supernatants were collected. Posterior eye cup lysates were prepared by incubation of freshly-dissected posterior eye cups without from 3 mice of each genotype (3 eyes: n=3) in RIPA buffer on ice for 20 min followed by brief vortexing at 5 min intervals. Tissue lysates were collected by centrifugation at 13000 rpm for 15 min at 4^0^ C. Protein concentration was quantified using the Bradford method (Bradford 1976), samples were resolved on SDS-PAGE, immunoblotted, and immunoreactive signals detected using an ECL imager^TM^ system (Thermo Fisher Scientific). Antibodies against clusterin (12289-1-AP) (Ma et al. 2018), vitronectin (15833-1-AP) (Tougan et al. 2018) and HTRA1 (55011-1-AP) were obtained from Proteintech (Ochiai et al. 2019). ADAM9 antibody was purchased from R and D systems (AF949-SP) (Delarasse et al. 2011) Beta-actin antibody (8H10D10) and anti-tubulin antibody (2128P) were obtained from Cell Signaling Technology. Anti-V5 Tag antibodies (ab9116) (He et al. 2019) CTRP5 rabbit polyclonal CTRP5 antibody (ab36893) and CTRP5 monoclonal antibody (Mandal et al. 2006b) and C3 antibody (ab17453) (Nakamura et al. 2017) were obtained from Abcam. All primary antibodies were used at a dilution of 1:1000 for immunoblotting. HRP conjugated secondary antibodies were purchased from SANTA CRUZ biotechnologies and used at a dilution of 1:10000. Chemiluminescence was detected using [Pierce ECL Western blotting substrate](https://www.thermofisher.com/order/catalog/product/32106)^[TM](https://www.thermofisher.com/order/catalog/product/32106)^ [(Thermo Fisher Scientific; 32106). The antibodies to CTRP5 and C3 have been validated using tissue lysates from respective knockout mice. The antibody to the V5 tag was validated using an untransfected control.](https://www.thermofisher.com/order/catalog/product/32106)

**Quantitative PCR**

For preparation of total RNA from posterior eye cups devoid of neuro-retina, eye cups from 4 mice (8 eyes; n=8) were dissected using a dissecting microscope, and initially stored at 4^o^ C in stabilization solution, RNAlater^TM^ (Thermo Fisher Scientific; AM 7020). Total RNA was isolated using an RNA extraction kit (Qiagen; 74104). Reverse transcription was performed using Superscript Reverse Transcriptase III (Thermo Fisher Scientific; 18080093). qRT-PCR was performed to analyze the expression of mouse *Ctrp5* and *Htra1* as described previously (Mandal et al. 2006a) using: forward (5’GTAGCGACGCCAAGACCTAC3’) and reverse (5’CTTCTCCACCACATCAGCAA3’) primers to analyze *Htra1;* forward- (5’GCTTGCAGTTTGATCTTGTC3’) and reverse (5’GGTTCACTGTGTTTTAAGCG3’) primers to analyze *Ctrp5* expression and the PCR products were validated by Sanger sequencing. Expression of Gapdh were used as control to normalize *Ctrp5* and *Htra1* expression*.*  Comparative *ct* method was used to calculate the expression of specific genes normalized to *Gapdh.*  Mean (±SEM) relative expression levels were calculated by analyzing at least three independent samples with replica reactions and the values were expressed graphically as relative mRNA compared to expression of *Gapdh.*

**Immunohistochemistry**

Staining for CTRP5 and HTRA1 in mice retina was performed on eye frozen cryosections. For CTRP5 staining, rabbit polyclonal CTRP5 antibody (Abcam; ab36893) was used at a dilution of 1:200. For HTRA1 staining, rabbit polyclonal HTRA1 antibody (Proteintech; 55011-1-AP) was used at a dilution of 1:500. Immunohistochemistry was performed using standard procedures and images were captured by confocal microscopy (Nikon A1R HD25). The extent of immunostaining was quantified using imageJ software (NIH).

**Proteomic analyses**

Immediately following euthanization, eyes were enucleated and the lens and retinas were removed. Phosphate buffered saline (50 µl) was added to the eye cup and immediately frozen on dry ice and stored at -80^o^ C until needed. The eyes were thawed and 50 µl PBS containing 5 mM EDTA was added. The RPE cells were removed by repeated washing with PBS/EDTA until the Bruch’s membrane was free of RPE cells. BM-Ch was dissected from each eye cup. Samples were composites of BM-Ch from 4 or more mice and up to 4 samples of each genotype were analyzed. Proteins were solubilized in 8M urea/2% SDS/5 mM EDTA by pestle homogenization. The samples were centrifuged and SDS gel sample buffer was added; samples were re-homogenized and left at room temperature for 1 h. Proteins were run 1 cm into a 10% SDS PAGE gel. The proteins in the entire lane were in-gel digested with trypsin (0.02µg/µl). Peptides were separated using a 4 h liquid chromatography gradient and masses were determined on a Q Exactive Plus Mass Spectrometer (Thermo Fisher Scientific) coupled to a nano ACQUITY UPLC System (Waters). Liquid chromatography tandem mass spectrometry (LC-MS/MS) was performed at the Proteomics and Metabolomics Facility, Wistar Institute. The proteins in the SDS PAGE gel were in-gel digested with trypsin and injected onto a Waters UPLC Symmetry trap column. Peptides were separated by reversed phase HPLC using a C18 nanocapillary analytical column and a 4 h gradient. MaxQuant 1.6.0.16 was used to identify peptide sequences (Cox and Mann 2008) MS/MS spectra were searched using the Uniprot mouse protein database (October 2017).

**Statistical analysis**

All the experiments performed in this study were repeated at least three times and the data was initially analyzed using Shapiro-Wilk test to check the normality and upon confirmation of normal distribution in the data sets, data was further analyzed using two-tailed, independent student t-test. The data are presented as mean± standard deviation. NS indicates non-significance in the comparison of two data sets. *p*-values were calculated using Graph pad Prism software version 6 (La Jolla, CA, USA). The *p* values less than 0.05, 0.01 and 0.001 are indicated with *, ** and ***, respectively.

**Supplementary Figure S1: Verifying CTRP5 as a bait for the Y2H system - test for autoactivation and toxicity**

WT-CTRP5 dimerizes (with moderate strength) and weakly interacts with MFRP in the Y2H assay. Full-length WT-CTRP5 and MFRP were fused to the binding domain (BD) or the activation domain (AD) of GAL4 to form bait and prey constructs, respectively, and used in Y2H experiments. Yeast cells carrying the proper combination of bait and prey plasmids were plated on semi-selective and selective media supplemented with 3-AT (1, 2 or 5 mM).

**Supplementary Figure S2: Comparison of Elastase activity assay of HTRA1 in the presence of purified WT-CTRP5 protein obtained from 2 different sources**

HTRA1 activities observed with WT-CTRP5 purified in house (black bars) or obtained commercially (grey bars).

**Supplementary Figure S3:**

Amido black staining of nitrocellulose membrane with purified fractions of WT-CTRP5 and S163R-CTRP5 proteins. BL 21 star (DE3) bacterial cells expressing WT or S163R-CTRP5 were lysed and the resultant supernatants were also analyzed along with the purified proteins. Bands at approximately 26 KDa represent purified CTRP5 protein.

**Supplementary Figure S4:**

Graphical representation of area of staining of HTRA1 and CTRP5 near sub-RPE region. Image J software (NIH) was used to measure the extent of staining. Three independent measurements of 100µM region was taken from different areas of the image.

**Supplementary Figure S5:** Hematoxylin and Eosin staining of retinal sections from *Wt* ,*Ctrp5^S163R/wt^* and *Ctrp5^S163R/S163R^* mice aged 8-11 months.

**Supplementary Table S1:** Bacterial and yeast strains and plasmids used in this study

**Supplementary Table S2:** Mass intensities of peptides identified in *Wt*, *Ctrp5^S163R/wt^* and *Ctrp5^S163R/S163R^* identified during mass spectrometric analysis. Peptide length and amino acid sequence identified was also indicated.

Bradford MM (1976) A rapid and sensitive method for the quantitation of microgram quantities of protein utilizing the principle of protein-dye binding Anal Biochem 72:248-254

Cox J, Mann M (2008) MaxQuant enables high peptide identification rates, individualized p.p.b.-range mass accuracies and proteome-wide protein quantification Nat Biotechnol 26:1367-1372 doi:10.1038/nbt.1511

Delarasse C, Auger R, Gonnord P, Fontaine B, Kanellopoulos JM (2011) The purinergic receptor P2X7 triggers alpha-secretase-dependent processing of the amyloid precursor protein J Biol Chem 286:2596-2606 doi:10.1074/jbc.M110.200618

Gietz RD, Woods RA (2002) Transformation of yeast by lithium acetate/single-stranded carrier DNA/polyethylene glycol method Methods Enzymol 350:87-96

He Y et al. (2019) Androgen signaling is essential for development of prostate cancer initiated from prostatic basal cells Oncogene 38:2337-2350 doi:10.1038/s41388-018-0583-7

Ma B et al. (2018) Proteomic analysis of rat serum revealed the effects of chronic sleep deprivation on metabolic, cardiovascular and nervous system PLoS One 13:e0199237 doi:10.1371/journal.pone.0199237

Mandal MN et al. (2006a) Spatial and temporal expression of MFRP and its interaction with CTRP5 Invest Ophthalmol Vis Sci 47:5514-5521 doi:10.1167/iovs.06-0449

Mandal MN et al. (2006b) CTRP5 is a membrane-associated and secretory protein in the RPE and ciliary body and the S163R mutation of CTRP5 impairs its secretion Invest Ophthalmol Vis Sci 47:5505-5513 doi:10.1167/iovs.06-0312

Nakamura K, Kusama K, Bai R, Ishikawa S, Fukushima S, Suda Y, Imakawa K (2017) Increase in complement iC3b is associated with anti-inflammatory cytokine expression during late pregnancy in mice PLoS One 12:e0178442 doi:10.1371/journal.pone.0178442

Ochiai N et al. (2019) Murine osteoclasts secrete serine protease HtrA1 capable of degrading osteoprotegerin in the bone microenvironment Commun Biol 2:86 doi:10.1038/s42003-019-0334-5

Stanton CM et al. (2017) Novel pathogenic mutations in C1QTNF5 support a dominant negative disease mechanism in late-onset retinal degeneration Sci Rep 7:12147 doi:10.1038/s41598-017-11898-3

Tougan T et al. (2018) Molecular Camouflage of Plasmodium falciparum Merozoites by Binding of Host Vitronectin to P47 Fragment of SERA5 Sci Rep 8:5052 doi:10.1038/s41598-018-23194-9
